# Fragile preferences: A deep dive into order effects in large language models

**DOI:** 10.1093/pnasnexus/pgag246

**Published:** 2026-08-11

**Authors:** Haonan Yin, Shai Vardi, Vidyanand Choudhary

**Affiliations:** Department of Information Systems and Business Analytics, Ivy College of Business, Iowa State University, Ames, IA 50011, USA; Department of Information Systems, Muma College of Business, University of South Florida, Tampa, FL 33620, USA; Department of Information Systems, Paul Merage School of Business, University of California, Irvine, Irvine, CA 92697, USA

**Keywords:** large language models, position bias, fragile preferences, preference distortion

## Abstract

Large language models (LLMs) are increasingly deployed in decision-support systems for high-stakes domains such as hiring and university admissions, where choices often involve selecting among competing alternatives. While prior work has noted position biases in LLM-driven comparisons, these biases have not been systematically analyzed or linked to underlying preference structures. We present the first comprehensive study of position biases across multiple LLMs and two distinct domains: resume comparisons, representing a realistic high-stakes context, and color selection, which isolates position effects by removing confounding factors. We find strong and consistent order effects, including a quality-dependent shift: when all options are high quality, models favor the first option, but when quality is lower, they favor later options. We also identify a previously undocumented bias: a *name bias*, where certain names are favored despite controlling for demographic signals. To separate superficial tie-breaking from genuine distortions of judgment, we extend the rational choice framework to classify pairwise preferences as robust, fragile, or indifferent. Using this framework, we show that order effects can lead models to select strictly inferior options. These results indicate that LLMs exhibit distinct failure modes not documented in human decision-making. We also propose targeted mitigation strategies, including a novel use of the temperature parameter, to recover underlying preferences when order effects distort model behavior.

Significance statement As large language models (LLMs) are increasingly used in high-stakes decision-making, their susceptibility to biases poses serious risks. We extend the rational choice model to distinguish robust from fragile preferences and show that order effects can reverse true preferences, leading LLMs to recommend strictly inferior options. We identify a quality-dependent shift in order effects, and a bias favoring certain names, neither of which have been reported in humans, highlighting that detection and mitigation strategies developed for human decision-making are insufficient for LLMs. To address this, we propose LLM-specific mitigation strategies, including a novel use of the temperature parameter to recover underlying preferences obscured by order effects.

## Introduction

Large language models (LLMs) are becoming increasingly integrated into high-stakes decision-support systems. For instance, in healthcare, LLMs are being used to assist in differential diagnosis and support triage decisions, where biases could have life-altering consequences (1); in finance, LLMs support fraud detection and automated customer service, where biases could lead to significant economic harm (2); in hiring, biases could systematically exclude qualified candidates (3). In such contexts, ensuring fairness and transparency is not just an ethical imperative but often a legal requirement, as recent regulations emphasize algorithmic accountability and nondiscrimination (4–6).

While studies have found that LLMs outperform humans in many domains such as social situational judgments, diagnosis, prediction, and persuasion (7–10), a growing literature highlights substantial risks associated with deploying these systems in real-world decision contexts. In particular, LLMs can exhibit systematic biases related to gender, race, and other demographic attributes (11–13), which can be subtle and, in some cases, even stronger than those observed in human decision-making (14). Beyond demographic bias, recent work documents linguistic stereotyping based on dialect (15) as well as cultural favoritism toward groups that are dominant within a given language context (16), raising concerns about fairness across diverse populations. Other work has documented sensitivity to prompt framing and contextual cues, which can lead to inconsistent or unstable decisions across otherwise equivalent inputs (17–19). Because these systems are increasingly embedded in automated pipelines, even small contextual effects may propagate into large-scale downstream outcomes.

The impact of these issues is further compounded by the interaction between LLM outputs and human decision-makers. Studies show that LLM-generated recommendations can increase user overconfidence and amplify existing human biases (20, 21), with downstream effects in which humans adopt or inherit AI-induced biases (22). More broadly, reliance on LLMs can lead to forms of cognitive surrender, where users defer to models’ outputs even when they are flawed (23). Together, these findings highlight that LLM outputs are not only subject to bias but can also shape and reinforce human decision-making in ways that may degrade outcomes in practice.

In many real-world decision-making settings, LLMs are used to compare alternatives, requiring models to choose among or rank competing options rather than evaluate each in isolation. Such comparisons arise, for example, in medical triage (24), LLM-as-a-judge frameworks (25), and hiring decisions (26). While alternatives could in principle be evaluated independently using absolute scores, a growing body of work advocates for pairwise or setwise comparisons, arguing that relative judgments are more consistent, robust, and aligned with human evaluation processes (27–30). However, although comparative judgments can improve reliability relative to evaluating items independently, they implicitly assume that preferences are invariant to irrelevant aspects of presentation, such as the order in which alternatives are shown. This makes them inherently sensitive to contextual and positional effects. In human decision-making, the order in which alternatives are presented can systematically influence judgments, as documented in extensive behavioral research on primacy and recency effects (31–36), anchoring (37, 38), and contrast effects (39, 40). When several similar alternatives are arranged in an array, people tend to choose the central options (41–43). Preferences can also be shaped by the composition of the choice set, for example via decoy effects (44–46). These biases lead to instability across equivalent presentations and can undermine consistency and lead to irrational preferences.

Recent studies show that LLMs are vulnerable to similar biases. Order effects have been documented in multiple-choice settings (47, 48), classification tasks (49, 50), conversational summarization (51), and pairwise evaluations (30, 52). Other context-related effects, such as anchoring and decoy effects, have also been observed (17, 53). Sensitivity to prompt ordering has also been observed in reasoning tasks (54, 55). Yet this literature remains largely descriptive: we know position biases exist, but not how different contexts influence them, the extent to which they are model-specific, or how they interact with other biases. It is also unclear whether they reflect tie-breaking or true preference distortions, or how they differ from position biases in humans.

In this work, we systematically investigate positional biases in LLMs and their implications for comparative decision-making.^a^ We uncover novel behavioral patterns, demonstrate that these effects can meaningfully distort underlying preferences (which we operationalize using random utility theory), and identify important differences from human biases. We examine these effects across five LLM families, comprising nine widely used models, and evaluate them in two fundamentally different domains: hiring decisions and children’s room color selection. The hiring scenario is a high-stakes application with a concrete link to existing literature on LLM biases. In contrast, color selection is a low-stakes, previously unexplored application that is minimalistic, neutral, and easily replicable. Because it is largely free of other well-documented biases such as gender or race, it allows us to isolate position effects more precisely than in complex domains. For each domain, we conduct experiments using four quality tiers. We uncover a consistent, quality-dependent pattern in pairwise comparisons across both domains: when both options are of relatively high quality, LLMs tend to favor the first candidate or color; when the options are of lower quality, they tend to favor the second. Although the trends are similar across models, what constitutes “high” and “low” quality may vary across models. In pairwise resume comparisons, for instance, Llama 3 8B favors the first candidate in the top two tiers and the second in the bottom two, while GPT-4o-mini favors the first candidate in the top tier, and the second in the bottom three. This quality-dependent shift appears to be a distinct pattern in LLM decision-making that, to our knowledge, has not been documented in human or prior LLM studies.

The pattern extends to triplewise comparisons: when all three options are high-quality, LLMs tend to prefer the first option, whereas for lower-quality options, they favor later ones. We further show that position biases are, for the most part, stronger than gender biases in our experimental settings, and uncover a significant and previously undocumented source of distortion: a bias favoring certain names over others. These patterns suggest that LLMs are not merely inheriting human-like heuristics from their training data but are manifesting new failure modes with distinct underlying mechanisms. We further find that these effects persist across languages (Mandarin Chinese and Hebrew), even though the underlying preferences are language-dependent.

In addition to these identified patterns, we highlight a conceptual distinction that has been largely overlooked in prior work on order effects in both humans and LLMs. Although rarely distinguished in prior work, position biases can arise from two fundamentally different phenomena: tie-breaking heuristics and true preference distortions. Tie-breaking occurs when the agent (human or AI) has no clear preference and defaults to a fixed positional rule (e.g. always guessing option C on a multiple-choice test). In contrast, a distortion occurs when the order of presentation alters an otherwise strict preference. This distinction is critical for interpreting observed order effects. While tie-breaking reflects indifference and is relatively benign, preference distortion implies that the model may select inferior options due solely to presentation order. Without distinguishing between these mechanisms, observed position effects may be misinterpreted as harmless randomness when they in fact reflect systematic distortions of underlying preferences.

Disentangling these mechanisms is difficult, especially in domains where ground-truth preferences are ambiguous. Many prior studies suggest that order effects emerge mainly when options are similar (30, 47, 56), consistent with the tie-breaking view. However, we show that position effects in LLMs can cause distortions that lead to genuine preference reversals. In other words, even though the LLM strictly prefers option *a* to option *b*, it might nevertheless choose option *b* when the two are presented in a certain order. To address this, we introduce a simple yet effective mitigation strategy: querying the model numerous times at higher temperature settings.

At low temperatures, models’ outputs are nearly deterministic. Assume that an LLM prefers option *a* to option *b* but there is a primacy bias strong enough to reverse this preference. Then, when the model is given a choice between *a* and *b* at a low temperature, it will always select the first option presented. Here, position biases can fully determine which option is selected, masking the model’s underlying preferences.

When the temperature parameter (denoted *T*) increases, the models’ outputs become more stochastic; repeated sampling shows more variation. While T=0 produces (nearly) deterministic outputs, higher values such as T=1 yield greater randomness. By repeatedly querying the model at higher temperatures and examining selection frequencies across permutations, we can recover the model’s underlying preference structure even when lower temperatures obscure it.

To formalize the preference structure, we extend the classical rational choice framework, which represents preferences using the binary relations a≻b (strict preference) and a∼b (indifference). We introduce a refined notation that distinguishes between two types of strict preferences. We write a≫b to indicate that the preference for *a* over *b* is *robust* and stable across contexts. In contrast, a≽b denotes a *fragile* preference: a genuine but unstable preference that may be reversed by order effects. The distinction between fragile and robust preferences is important because fragile preferences imply that LLM decisions may depend on arbitrary aspects of prompt structure leading the model to make suboptimal decisions.

We define these preferences more formally within our setting using random utility theory (57), where one defines preferences using selection probabilities. Let Pr[a∣{a,b},t] denote the overall probability of selecting *a* when both permutations (a,b) and (b,a) are presented an equal number of times at temperature *t*. Intuitively, if Pr[a∣{a,b},t]=0.5 for all temperatures *t*, then the model is indifferent between *a* and *b*. If there exists some temperature *t* such that Pr[a∣{a,b},t]>0.5, then the model strictly prefers *a* to *b*. We note that we assume there do not exist $t_{1}$ and $t_{2}$ such that $\text{Pr}[a|\{a,b\},t_{1}]>0.5$ and $\text{Pr}[a|\{a,b\},t_{2}]<0.5$, i.e. we rule out preference reversals across temperatures. This is consistent with the role of temperature in language models, which adjusts the randomness of outputs while generally preserving the relative likelihood of alternatives.

At T=0, if the model’s output were perfectly deterministic, there would be only two possible behaviors: (i) the same option is selected in both orders, giving selection probabilities of {0,1} and (ii) the model consistently picks one position (e.g. the first option), so each option is chosen half of the time, giving {0.5,0.5}. In practice, T=0 is not perfectly deterministic; we use “≈” to indicate probabilities close to, but not exactly, 0, 0.5, or 1.^b^ Preferences are classified as follows:

- If Pr[a|{a,b},0]≈1 and Pr[a|{a,b},1]>0.5 then a≫b.
- If Pr[a|{a,b},0]≈0.5 and Pr[a|{a,b},1]>0.5 then a≽b.
- If Pr[a|{a,b},0]≈0.5 and Pr[a|{a,b},1]=0.5 then a∼b.

Preferences that do not fit into any of these cases (for example, if Pr[a∣{a,b},0]≈1 while Pr[a∣{a,b},1]≈0.5) are deemed *inconsistent*; we do not observe such cases in our experiments.

Simply put, if the model selects the same option (say *a*) when queried with (a,b) and (b,a) at T=0, then a≽b; the model robustly prefers *a* to *b*. If the model’s choice flips when the presentation order is reversed at T=0, then the model may either be indifferent between the two options (a∼b) or have a fragile preference for one of them (a≽b or b≽a). These cases cannot be distinguished based on T=0 alone.

To differentiate between them, we query the model at T=1, using an equal number of each permutation. If one option is selected more frequently—for example, if Pr[a∣{a,b},1]>0.5—we interpret this as a fragile preference (a≽b), meaning that the order-dependent reversal at T=0 reflects a genuine preference distortion. If both options are selected with approximately equal frequency (i.e. Pr[a∣{a,b},1]≈0.5), we interpret this as indifference (a∼b), in which case the observed order effect is consistent with tie-breaking. Taken together, this provides a simple procedure: query at T=0 to detect order sensitivity, and, when present, use repeated sampling at higher temperatures to determine whether the effect reflects indifference or a distorted preference.

In practice, distinguishing between fragile preference and indifference may be difficult when Pr[a∣{a,b},1] is close to 0.5. However, in such cases the underlying preference is necessarily weak, and the distinction has limited practical impact. In practice, this suggests fixing a reasonable number of samples and testing whether selection frequencies differ significantly from 0.5; if not, the underlying preference is weak and the distinction between fragile preference and indifference is unlikely to be consequential.

When order effects at low temperatures obscure underlying preferences, increasing temperature serves as a diagnostic tool, revealing these latent preferences through repeated sampling. By introducing controlled stochasticity, higher temperatures allow us to estimate the underlying preference distribution rather than observe only a single, potentially biased outcome. As a result, in such settings, higher temperatures can improve preference detection accuracy, contrary to the prevailing understanding that greater accuracy is attained at lower temperature settings (58, 59).

Our findings suggest that comparative decisions in LLMs are shaped by a combination of familiar and novel biases, and that these biases can systematically distort underlying preferences. Importantly, these distortions are not merely artifacts of indifference or randomness: they often reflect genuine preference reversals driven by presentation order. This has practical implications for how LLM-based comparisons should be interpreted and used. In particular, positional effects can outweigh demographic biases and cannot be reliably mitigated by simple strategies such as averaging or randomizing presentation. Instead, careful analysis of selection patterns across permutations and temperatures is required to recover and interpret underlying preferences.

## Results

We selected two domains to balance realism and control over confounding factors: resume screening and color selection. In the hiring domain, we evaluated three large language models: GPT-4o-mini, Claude 3 Haiku, and Llama 3 8B. In the more controlled color-selection setting, we examined position biases across nine widely used LLMs spanning five families: OpenAI (GPT-4o-mini and GPT-4.1-nano), Anthropic (Claude 3 Haiku and Claude Sonnet 4), Meta (Llama 3 8B and Llama 4 Scout), Google (Gemini 2.5 Flash and Gemini 3 Flash), and Alibaba (Qwen 3 32B).

As order effects are most likely when alternatives are similar in quality (30, 47), we created item sets close in quality and grouped them into four tiers: *Best*, *Good*, *Mediocre*, and *Weak* for resumes, and *Ideal*, *Fair*, *Plain*, and *Harsh* for colors. In the hiring domain, we used LLM-generated resumes for four professions (Mechanical Engineer, Registered Nurse, Journalist, and Real Estate Agent). In the color domain, we curated sets of wall paint colors grouped by suitability for a child’s room.

For each model and domain, we conducted exhaustive pairwise and triplewise comparisons, testing all permutations of item order. Prompts included “Select the strongest candidate” and “Which color is best for a kid’s room?” Each model was evaluated at multiple temperature settings. At T=0, models behaved with near-determinism, selecting the most likely answer. At T=1, responses varied across repeated queries, allowing statistical analysis of position effects; each T=1 response was treated as an independent sample from the model’s output distribution.

We define position biases as follows. A model exhibits a *primacy bias* if there exists a set of options such that the model disproportionately prefers whichever option is presented first. It exhibits a *recency bias* if there exists a set of options such that it disproportionately prefers whichever option is presented last. In triplewise comparisons, it exhibits a *centrality bias* if there exists a set of three options such that it disproportionately prefers whichever option is presented second. A model may exhibit more than one bias depending on the context.

In the main text, we present results from three representative models—GPT-4o-mini, Claude 3 Haiku, and Llama 3 8B. Full experimental details appear in the Materials and methods section and SI Appendices. The results for the remaining models are presented in SI Appendix K.

### Order effects in pairwise comparisons

**Figure 1:**
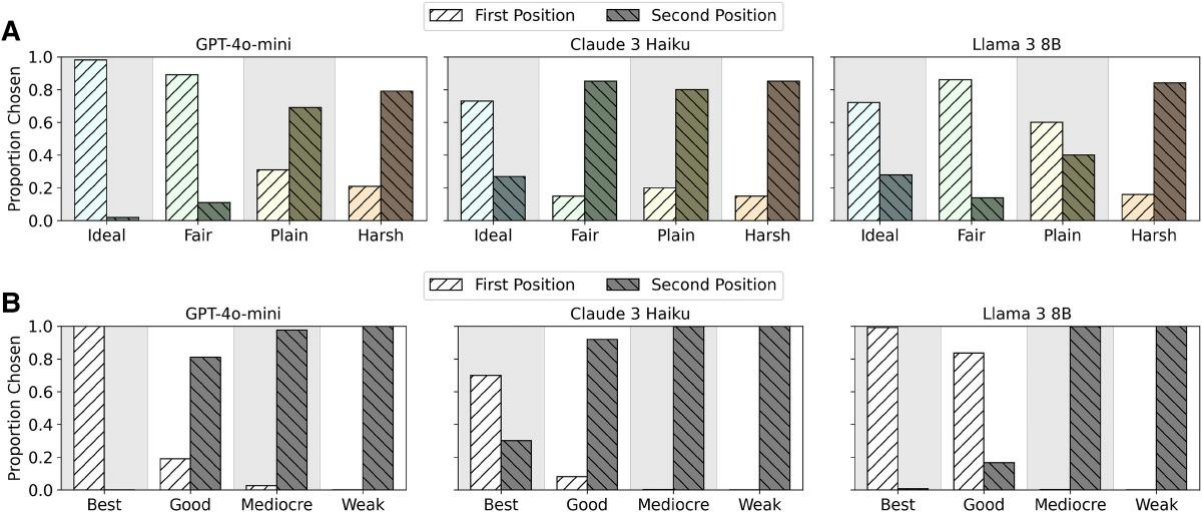
Position bias in pairwise comparisons at T=1. A) Proportion of color choices by position across four quality tiers in pairwise comparisons. Tier quality decreases from left to right: “Ideal” is the highest-quality tier and “Harsh” is the lowest-quality tier. It shows the effect of presentation order on color selection in pairwise comparisons for each quality tier. B) Position bias in pairwise resume selection, aggregated across professions. It shows aggregate positional effects in resume evaluations across professions. In both tasks, higher-quality options tend to exhibit a primacy effect, whereas lower-quality options tend to exhibit a recency effect, although the precise threshold separating them varies by model and domain. Full results for color comparisons are provided in SI Appendix C; results for resumes disaggregated by profession are provided in SI Appendix D.

We find a consistent, quality-dependent position effect across models and domains: higher-quality options tend to exhibit primacy effects, while lower-quality options exhibit recency effects. For instance, in color selection (Fig. 1A), GPT-4o-mini displayed a primacy effect in the top two tiers (Ideal and Fair) and a recency effect in the bottom two (Plain and Harsh). Claude 3 Haiku and Llama 3 8B showed a primacy effect in the top one and three tiers, respectively, and a recency effect in the bottom three and one tiers, respectively. In Fig. 1B, the resume selection results followed a similar overall pattern (though GPT-4o-mini and Claude 3 Haiku showed primacy only in the top tier, and Llama 3 8B in the top two tiers). This pattern suggests that position bias interacts systematically with perceived quality; position effects depend on the underlying preference structure.

Full statistics for these three models, including those for additional temperature settings, appear in SI Appendices C and D. Details for additional models are presented in SI Appendix K.

### Order effects in triplewise comparisons

**Figure 2:**
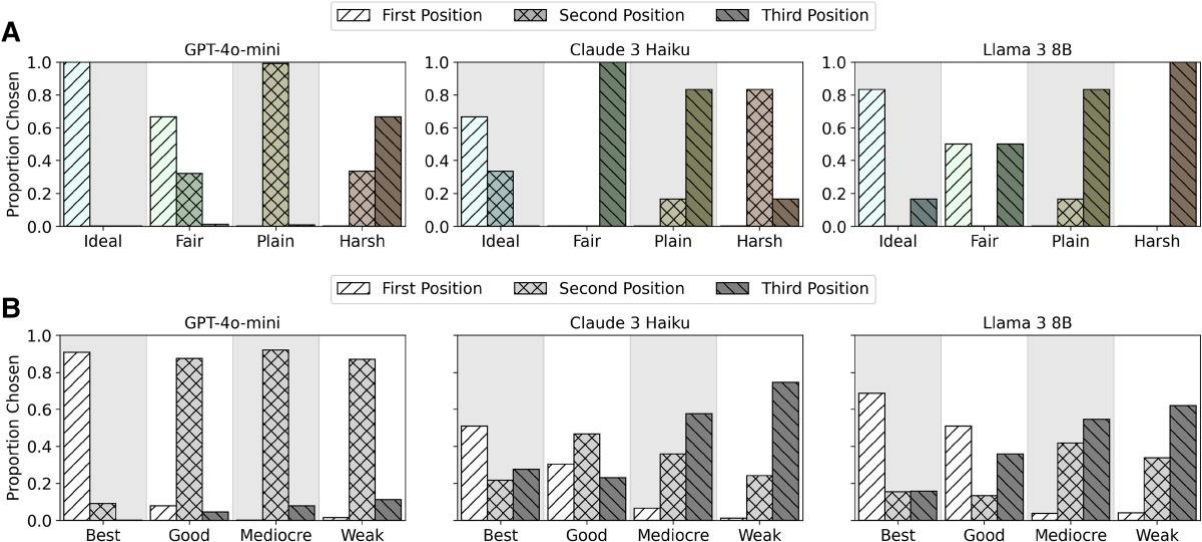
Position bias in triplewise comparisons at T=0. A) Proportion of color choices by position across four quality tiers in triplewise comparisons. It shows the effect of presentation order on color selection. B) Aggregate position bias in triplewise resume selection across four quality tiers and professions. It shows aggregate position effects in resume evaluations across professions. Additional results for color selection are provided in SI Appendix F, and resume-selection results disaggregated by profession are provided in SI Appendix G.

In triplewise comparisons, higher-quality tier(s) exhibited a primacy effect, consistent with the pairwise results. For lower-quality tier(s), all models favored later positions, though they differed in whether the second or third option was preferred. Specifically, GPT-4o-mini and Claude 3 Haiku sometimes favored the second (middle) position, exhibiting centrality bias. For example, at T=0 (Fig. 2A), GPT-4o-mini always selected the middle option for *Plain* colors, and Claude 3 Haiku did so 83% of the time for *Harsh* colors. In resume selection (Fig. 2B), GPT-4o-mini preferred the middle option in three out of the four tiers tested.

Several of the models showed simultaneous primacy and recency effects, resembling the serial position effects observed in humans (34, 60). Among colors in the *Fair* tier at T=0, Llama 3 8B chose the first and last positions equally often (50% each) and never selected the middle option. See SI Appendices F and G for additional details. Results from additional large language models are presented in SI Appendix K.

Across models and domains, position bias generally shifted toward later positions as quality declined, with the exception of Claude 3 Haiku in the color domain. Position biases were notably weaker in resume selection for Claude 3 Haiku and Llama 3 8B compared to color selection. As we show in the *Name Bias* section below, this attenuation is largely due to interference from strong preferences for specific names. These results show that position biases can manifest in more complex ways and suggest that they are not limited to simple heuristics, but can adapt to the structure of the choice set.

To explore whether position biases arise in larger choice sets, we conducted preliminary fourwise color comparisons for each model. Although we only evaluated three models, and a single quality tier per model, the results demonstrate that position biases can persist in more complex choice scenarios. For full details, see SI Appendix H.

### Cross-language robustness and preference shifts

We replicated the color-selection experiments in Mandarin Chinese and Hebrew, using GPT-4o-mini. The overall positional patterns remain consistent across languages: higher-quality options tend to exhibit primacy effects, while lower-quality options exhibit recency effects.

However, the specific options exhibiting these effects differ across languages. In English, blue tones (e.g. Aqua Mist, Soft Sky Blue) tend to exhibit primacy effects, while pastel tones (e.g. Gentle Coral, Buttercream Yellow) exhibit recency effects. In Hebrew, this pattern reverses: pastel tones exhibit primacy effects, while blue tones exhibit recency effects.

This difference reflects a shift in underlying preferences: in English, the model tends to prefer blue tones, whereas in Hebrew it prefers pastel tones. This shift is also evident in specific pairwise comparisons. For example, when choosing between Frosted Cyan and Transparent Ice Blue at T=0, GPT-4o-mini consistently selects Frosted Cyan when prompted in English but selects Transparent Ice Blue when prompted in Hebrew. When prompted in Mandarin Chinese, it displays a primacy effect: selecting the first option presented. These results suggest that models’ preferences are language-specific, and not absolute.

### Tie-breaking heuristics vs. true distortions

Do order effects reflect arbitrary tie-breaking, or genuine distortions of the model’s underlying preferences? Our notation allows us to distinguish between these cases in pairwise comparisons. For color selection, all but one of the thirty-six model-tier pairs satisfied Pr[a∣{a,b},0]≈0.5: at T=0; all models selected the same position at least 98% of the time for each tier. When the option selected is order-dependent at T=0 (i.e. Pr[a∣{a,b},0]≈0.5), exactly one of the following holds: a≽b, b≽a, or a∼b. In other words, the model might be indifferent between the two colors or have a fragile preference for one.

To determine whether the models were indifferent or had a fragile preference, we repeated the comparisons at T=1. If Pr[a∣{a,b},1]≈0.5, the model is indifferent (a∼b); if Pr[a∣{a,b},1]>0.5, the model prefers option *a* (a≽b). To build intuition as to why an increase in temperature can help distinguish between indifference and a true preference, consider a stylized example with von Neumann–Morgenstern (VNM) utilities.^c^

Suppose that there is a primacy boost δ=0.2; that is, the perceived utility of the first option is increased by 0.2. Assume first that the model is indifferent between the two options and assigns u(a)=u(b)=5. At T=0, presenting (a,b) yields *a* (since 5+0.2>5), and presenting (b,a) yields *b* (since 5+0.2>5). Consider now the case that (b≽a), and the model assigns u(a)=5,u(b)=5.1. At T=0, presenting (a,b) yields *a* (since 5+0.2>5.1), and presenting (b,a) yields *b* (since 5.1+0.2>5). In both cases, the choice is completely order dependent.

Now suppose T=1 adds uniform noise ε∼U[−0.4,0.4] to the first option’s utility (on top of the position bias).^d^ Then (a,b) yields *a* with probability 0.625 (P[5.2+ε≥5.1]=0.625), and (b,a) yields *b* with probability 0.875. Thus, *b* is significantly more likely to be chosen than *a* at T=1, revealing a strict (but fragile) preference for *b* over *a*, rather than indifference.

We conducted this experiment on our three representative models: GPT-4o-mini, Claude 3 Haiku, and Llama 3 8B. Consistent with this intuition, eight of twelve model-tier color pairs in our experiments showed a significant preference (P<0.05), and three of these showed overwhelming distortion ($P<10^{-6}$). We obtained similar results for resumes; in one case, one resume was 1.5 times more likely to be chosen than the other, indicating that order effects can lead to the consistent selection of inferior options—effects that are most pronounced at lower temperatures. This provides evidence that order effects often reflect genuine distortions of underlying preferences rather than arbitrary tie-breaking. For more details, see SI Appendix E.

### Interaction of position bias with other biases

In addition to order effects, LLMs exhibit other well-documented biases, such as preferences based on gender or identity. In this section, we examine how these biases interact with position effects. While gender bias in algorithmic decision-making is well documented, we also uncovered an additional bias: consistent preferences for specific individual names, even when those names were carefully selected to avoid known bias-triggering cues such as race and ethnicity. To our knowledge, such name-specific biases have not been documented in human decision-making under comparable conditions. Across all models, we found that both gender and name biases are generally weaker than the position effects, although in some cases they appear to compete with or modulate them.

#### Name bias

The position bias in triplewise color comparisons appears much stronger than in resume comparisons particularly for Claude 3 Haiku and Llama 3 8B (see Fig. 2A). This reduction in position bias appears to have been driven primarily by strong, competing biases for or against specific synthetic personas, especially individual names. Claude 3 Haiku, in particular, exhibited strong name-based preferences: it consistently favored certain names over others, regardless of resume content or position. For example, in direct comparisons, Claude 3 Haiku selected “Christopher Taylor” over “Andrew Harris” in 64% of the cases (82 out of 128 trials, binomial P=0.0019,h=0.29). These patterns emerged even though all names were drawn from a controlled set of generic Caucasian US identities, chosen to minimize racial, ethnic, and other socially marked biases that have been documented in LLMs (61, 62).

**Figure 3:**
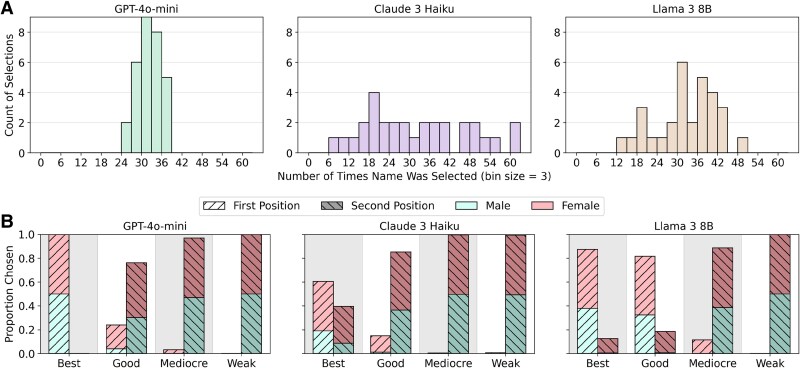
Interaction of order effects with other biases. A) Distribution of name-selection frequencies in triplewise comparisons. The *x*-axis represents the number of times a name was selected, and the *y*-axis shows how many names were selected that number of times. Claude 3 Haiku exhibits strong preferences for certain names, whereas GPT-4o-mini shows a relatively balanced selection pattern. For GPT-4o-mini, nine names were selected between 30 and 32 times, and all names were selected between 24 and 38 times. Additional results and details are provided in SI Appendix I. B) Gender and order effects at T=0 in pairwise resume comparisons. All models exhibit stronger biases for presentation order than for gender. Results by profession and temperature are provided in SI Appendix J.

This pattern is evident in Fig. 3A, which shows the distribution of selection frequencies for each name across triplewise comparisons. Claude 3 Haiku’s distribution is flat, indicating that a few names were chosen disproportionately often while others were rarely selected—consistent with strong and persistent name preferences. GPT-4o-mini, by contrast, displays a near-perfect normal distribution, suggesting minimal name-based bias in the triplewise setting. Llama 3 8B lies between these extremes, with moderate variation across names. Although GPT-4o-mini showed no signs of name bias in triplewise comparisons, we detected a small but statistically significant name preference in the pairwise setting. See SI Appendix I for full details.

#### Gender bias

Prior work on gender bias in algorithmic hiring is mixed: some studies report little to no bias (63), others find systematic preferences for male candidates (3, 64), while more recent evaluations suggest a tilt toward female candidates (26, 65) or context-dependent outcomes (12). Studies using pairwise evaluations have found a bias favoring female candidates (26, 52, 65), a pattern we also replicated: for example, GPT-4o-mini selected female candidates 54.8% of the time at T=0, with Claude 3 Haiku and Llama 3 8B showing similar trends.

While gender bias in algorithmic decision-making is an important topic in its own right, our primary goal is to examine how gender interacts with order effects. We found that gender effects were overshadowed by substantially stronger order biases. Across all three models, the candidate in the dominant position (first or second, as defined by same-gender comparisons) was selected at much higher rates. For GPT-4o-mini, this rate reached 92.8% in mixed-gender comparisons, compared to 93.6% in same-gender trials, suggesting that gender bias can at times counteract, but rarely override, the positional preference. Claude 3 Haiku and Llama 3 8B showed similar patterns, with position bias rates of 84.3 and 72.3% in mixed-gender comparisons, versus 87.6 and 89.2% in same-gender comparisons, respectively. Bootstrapped CIs confirm that order bias significantly exceeds gender bias across all models (95% CIs for $h_{\mathrm{order}}-h_{\mathrm{gender}}$ excludes 0 in all cases); see SI Appendix J for figures and additional details.

This difference between the effect sizes of the position and gender effects can be clearly seen in Fig. 3B, where gender effects manifest in two ways: a modest overall preference for female candidates, and the observation that nearly all instances of overcoming the positional disadvantage involve female candidates. Full statistics appear in SI Appendix J.

## Discussion

This study provides evidence of systematic position biases in LLM-driven comparisons, with methodological, theoretical, and practical implications. Across both color and hiring domains, we find that position biases are quality-dependent and strong: primacy effects dominate for high-quality options, while later options (second or third) are preferred for lower-quality choices. Because many applications rely on LLMs to rank, filter, or select among alternatives, even small systematic biases can propagate through decision pipelines and lead to consistently suboptimal outcomes.

Most prior research has not clearly distinguished between two possible sources of position effects: arbitrary tie-breaking and genuine preference distortion. Many studies have observed that order effects appear when the model is indifferent between the different options (30, 47, 56), and studies on position biases and the decoy effect often purposefully use alternatives of similar quality (17, 66). While some human studies have attempted to disentangle these explanations (67), conclusions remain limited due to humans’ limited ability to determine their “true” preferences (37) or compute expected utilities (68). Determining ground-truth preferences in human subjects is further complicated by contextual influences and shifts in preferences over time (69).

We leverage features of LLMs that are not available in human studies—the ability to repeat identical queries without memory carryover, and the temperature parameter—to distinguish tie-breaking from preference distortion. Using this methodology, we show that LLMs do not merely exhibit position effects due to indifference but also because position effects can be strong enough to reverse strict preferences. Although subtle, this distinction has important practical consequences: tie-breaking is relatively benign, whereas distortion can lead to selecting an inferior option purely because of the order of presentation.

Much of the work on LLM bias has centered on uncovering and correcting cognitive distortions, such as anchoring, confirmation bias, or gender stereotyping, that models acquire from human-generated training data (61, 70–72). These studies often argue that models inherit cognitive biases that are present in the data (73–75). Even when studies identify behavior that diverges from human norms (for instance, a female-candidate preference in resume-ranking tasks, where humans often favor male candidates), these findings are often interpreted through the lens of human cognitive biases (26, 65). In contrast, we uncover two failure modes that do not fit cleanly within standard accounts of human cognitive bias: position bias that varies systematically with option quality, and a name bias, even though the names were explicitly chosen to avoid known demographic associations.

These patterns raise new theoretical questions. Classical explanations for human order effects, such as memory limitations (76, 77), proactive inhibition (78), or heuristics (37), cannot readily explain our findings of quality-dependent bias shifts. One possible account, drawing on the long-established notions of reference points (60, 79) and contrast effect (39, 40) in behavioral decision theory, is that LLMs establish an implicit reference point from their training data and then evaluate each option in contrast to this baseline. Above-baseline options are inflated, leading to primacy, while below-baseline ones are penalized. However, it is not clear how to extend this explanation to biases observed in triplewise comparisons, in particular the centrality bias. Moreover, model-specific differences (e.g. centrality effects in GPT-4o-mini and Claude 3 Haiku but not Llama 3 8B) suggest that distinct bias mechanisms may arise across models, potentially due to differences in architecture or training, though the precise sources of these differences, especially in closed models, remain unclear. Together, these findings call for new theoretical frameworks that go beyond human cognitive models in explaining how biases emerge and propagate in artificial agents.

Our findings also carry practical implications for the deployment of LLMs in real-world decision systems. It is critical to not only monitor for human-like cognitive distortions inherited through training data, but also to proactively identify and mitigate novel failure modes that emerge from the models’ architectures or training dynamics. These uniquely artificial biases may be harder to anticipate precisely because they have no direct human analog. This underscores the importance of robust evaluation practices tailored to large language models.

We find that position biases are generally stronger than gender biases. This might be due to alignment efforts that explicitly target and reduce social biases like gender, while largely overlooking position biases. Nevertheless, because position effects currently overshadow gender-based biases, their mitigation is equally, if not more, critical.

However, standard mitigation strategies such as randomizing orders or averaging across queries (47, 50) do not fully eliminate order effects. Randomizing the presentation order in a single query merely randomizes which option benefits from the bias. Evaluating all permutations and taking the majority (or plurality) response also fails at T=0: in pairwise comparisons, fragile preferences lead to each option being selected equally often across permutations. As a result, these approaches do not recover the model’s underlying preferences and can mask systematic distortions. To address these effects, we propose two LLM-specific mitigation approaches:

1. *Perform repeated queries at a higher temperature to uncover suppressed preferences.* Prevailing wisdom suggests that responses at higher temperatures are less accurate (58, 59). This is true for robust preferences: if a≫b, option *a* will always be selected at T=0, while option *b* may be selected at T=1 due to stochasticity. Hence, queries at T=0 reliably recover robust preferences. However, position bias can completely mask fragile preferences at T=0, rendering them indistinguishable from true indifference. In such cases, increasing the temperature introduces controlled stochasticity that allows underlying preferences to emerge through repeated sampling. Thus, higher temperatures serve as a diagnostic tool: rather than reducing accuracy, they can improve preference detection by revealing latent preferences.
  Repeated querying also allows us to quantify preference strength. For example, if a model selects *a* over *b* 60% of the time at T=1, and *c* over *d* 80% of the time, we can infer that a≻b, c≻d, and that the latter preference is stronger. While our theoretical framework distinguishes between *robust*, *fragile*, and *indifferent* preferences, in practice preference strength varies continuously and can be estimated with high statistical confidence through repeated sampling. We note that this approach requires multiple rounds of inference and assumes that selection frequencies at T=1 provide a meaningful signal of underlying preference structure.
2. *Use position bias as a diagnostic signal.* Position bias itself can provide information about the underlying preference structure. For instance, a strong primacy effect may indicate that both options are of relatively high quality, while a strong recency effect may suggest that both are of lower quality. This interpretation follows from the observed interaction between quality and position bias. As a result, position effects can be used to guide downstream decisions, for example by triggering additional evaluation or human review when strong order effects are detected.

These strategies treat position bias not only as a problem to be corrected but also as a source of information. In practice, they can be incorporated into evaluation pipelines by running targeted high-temperature queries to detect fragile preferences, and by interpreting the magnitude and direction of position bias as indicators that guide further decision-making. Crucially, our experimental results show that these approaches can recover suppressed preferences and reduce the likelihood of selecting an inferior option due solely to presentation order.

Our results hold across two disparate domains, suggesting that they are inherent rather than domain-specific. While future training techniques may reduce such distortions, our findings suggest that positional sensitivity is currently widespread across models and can affect decision-making in systems that rely on comparative evaluation. Nevertheless, new architectures should be audited for order and identity biases. In high-stakes domains such as legal reasoning, medical diagnosis, or scientific peer review, bias-aware evaluation pipelines can combine model-driven diagnostics with human-in-the-loop checks. Finally, causal interpretability methods and adversarial probing could illuminate the internal mechanisms that give rise to preference distortions, paving the way toward bias-robust LLM design and deployment. Whether position biases can be fully eliminated remains an open question, and one that we believe warrants dedicated future investigation, particularly given the consistency of these effects across architectures and training regimes. These findings call for new theoretical frameworks that go beyond human cognitive models in explaining how biases emerge and propagate in artificial agents, particularly in systems deployed for automated or assisted decision-making.

## Materials and methods

### Resume and color generation and selection

As order effects are more pronounced when the options are of comparable quality, we generated three options for each of four quality tiers of colors and resumes. For colors, we denoted these tiers by *Ideal*, *Fair*, *Plain*, and *Harsh*; for resumes, by *Best*, *Good*, *Mediocre*, and *Weak*. We chose different names for the tiers in each domain so as not to imply an equivalence between them (i.e. *Plain* colors are not necessarily comparable to *Mediocre* resumes in terms of quality).

We first generated the candidates for each tier. For the resumes, we had GPT-4o-mini generate nine sets of four resumes of decreasing quality for each of four professions: Mechanical Engineer, Registered Nurse, Journalist, and Real Estate Agent. We removed the personal information from the resumes. The resume generation prompts are shown in SI Appendix B. For colors, we asked each model to generate lists of colors that they “thought” were comparable.

To validate the tier labels, we compared all items in each tier with all items in the next lower tier (highest vs. second-highest, second-highest vs. third-highest, and third-highest vs. lowest). We verified that, in each case, the higher-tier colors/resumes were more likely to be selected (one-sided binomial test, null hypothesis: P=0.5) with *P*-values of at most 0.001.

For each model and quality tier, we performed triplewise comparisons at T=0 among the candidate options and selected three options such that each was selected in at least one of the six possible permutations. To select the colors for pairwise comparisons in each tier, we evaluated all three possible pairs of options chosen for the triplewise comparisons. For each pair, we presented the options in both permutations at T=0. We selected a pair in which a different option was chosen for each permutation. We note that our selection methodology led to the fact that we did not necessarily use the same resumes or colors for the three models.

One might instead attempt to elicit preferences directly, by querying the model for equally preferred alternatives or by introducing an explicit “tie” response. We evaluated both approaches. When asked to provide colors that were equally good, models nonetheless exhibited a clear preference for one color in subsequent queries. Similarly, introducing a tie option did not eliminate position effects.

For the personal information, we generated 30 fictional personas, using 15 common American Caucasian last names, male first names and female first names, selected from the list of the most popular names in the US as tracked by the SSA (80). We paired each first name with a random surname and state, and randomly extrapolated to a complete profile. For more details, see SI Appendix B.

### Pairwise comparisons

For each pair, we presented the model with both orders to isolate position effects. The exact prompts used are included in SI Appendices A and B.

For resumes, we selected four male names and four female names and performed exhaustive same-gender comparisons. That is, for each pair of names *i* and *j*, we compared resume 1 with *i*’s personal information to resume 2 with *j*’s information, and vice versa. This yielded a total of 768 pairwise comparisons per model at each temperature (4 professions × 4 tiers × 2 genders × 6 name pairs × 2 name-resume assignments × 2 presentation orders). For colors, we repeated the prompt for each permutation 50 times at each temperature T∈{0,0.5,1}. Results for the hiring setting are presented in SI Appendix D, while results for the color context are provided in SI Appendices C and K.

For the cross-gender comparisons, we used the same resumes and personal information as the same-gender comparisons and compared all combinations of male and female names. That is, for each male name *i* and female name *j*, we compared resume 1 with *i*’s personal information to resume 2 with *j*’s information, and vice versa. This yielded 1,024 additional pairwise comparisons per model at each temperature (4 professions × 4 tiers × 4 female names × 4 male names × 2 name-resume assignments × 2 presentation orders). Detailed results on the interaction between gender effects and position bias are presented in SI Appendix J.

To verify that the observed name bias stemmed specifically from the name rather than other personal information (e.g. address, email host), in SI Appendix I, we performed additional comparisons after swapping the names while keeping the remaining details fixed.

To assess the cross-linguistic generalizability of the observed positional effects, we replicated the color comparison experiments across multiple languages, including Mandarin Chinese and Hebrew. English and Chinese employ a left-to-right writing and reading direction, whereas Hebrew employs a right-to-left direction. All color names and prompts were translated accordingly. The pairwise and triplewise comparison prompts, as well as detailed results, are reported in SI Appendix L.

### Triplewise comparisons

For each resume triple, we randomly assigned the 15 male and 15 female names (and their associated personal information) to the three resumes, yielding 5 distinct triples per gender. We then presented all six permutations of each triple to each LLM, asking the model to select the strongest candidate; the exact prompt is provided in SI Appendix B.

In total, we conducted 960 triplewise comparisons per model at each temperature (4 professions × 4 tiers × 2 genders × 6 permutations × 5 sets of triples). For colors, we repeated the prompt for each of the six permutations 40 times at each temperature, yielding 240 prompts per tier and 960 prompts per model at each temperature. The color comparison prompts are provided in SI Appendix A, and their versions in different languages are shown in SI Appendix L.

### Statistical tests

We evaluated position order effects in pairwise comparisons using two-tailed binomial tests, assessing whether the proportion of selections for the first versus second position significantly deviated from the null hypothesis of no position bias (equivalent to P=0.5). As all other effects are controlled for, a statistically significant deviation indicates that the model’s choices were systematically influenced by presentation order. We report effect sizes using Cohen’s *h*, a standardized measure of deviation from the expected proportion. All *P*-values and effect sizes are provided in the SI Appendices C and D.

For triplewise comparisons, we used chi-square goodness-of-fit tests to evaluate whether the distribution of selections across the three positions departed from a uniform distribution (i.e. each position selected with probability one-third under the null hypothesis of no bias). Significant deviations indicate systematic positional preferences. Effect sizes for these tests are reported using Cramér’s *V*. Full statistical results appear in SI Appendices F and G. We did not perform statistical tests for comparisons at T=0 as the results are almost deterministic.

To compare the relative strength of gender and position biases, we used the same dataset and performed nonparametric bootstrapping. For each of 10,000 resamples, we computed effect sizes for both gender and position bias within the same matched examples. The resulting bootstrap distribution allowed us to assess the consistency and magnitude of the difference, confirming that order effects consistently exceeded gender effects across resamples.

## Supplementary Material

pgag246_Supplementary_Data

## Data Availability

Replication archive with code and data is available at Open Science Framework at https://osf.io/59df6/. All other data are included in the SI Appendix.

## Notes

a These should be distinguished from a different form of position bias arising from interactions between attention mechanisms and positional embeddings (81). In contrast, we study biases in which models favor alternatives based on their position within a set of choices.

b Because T=0 does not always guarantee perfectly deterministic outputs, we fixed the random seed whenever preliminary runs exhibited non-negligible variability at T=0. We classify a frequency as approximately equal to one of 0, 0.5, or 1 if it falls within 0.05 of that value; in practice, all models tested deviated by at most 0.02 from the relevant benchmark value.

c A VNM utility function is any function that represents a preference relation consistent with the VNM axioms. See (82) for a formal definition. We use VNM utilities only for intuition; we do not claim that the models’ preferences can be mapped to such a function or that they satisfy the axioms of rationality.

d Noise can be assumed to be added to only one position without loss of generality.

## Preprints

A preprint of this article is available at arXiv:2506.14092.
