# Supplementary material for: Fragile preferences: A deep dive into order effects in large language models
Source: PNAS Nexus. 2026 Aug 11;5(8):pgag246. doi: 10.1093/pnasnexus/pgag246 (PMC13459133; doi:10.1093/pnasnexus/pgag246)
Supplement: Supplementary file 1 [file pgag246_supplementary_data.pdf]

Fragile Preferences: A Deep Dive into Order Effects in Large Language Models

Haonan Yin* Shai Vardi† Vidyanand Choudhary‡

July 12, 2026

This PDF file includes Supporting Information (SI):

- Color Comparison Sets and Prompts: Appendix A.
- Resume Comparison Sets and Prompts: Appendix B.
- Pairwise Color Comparisons: Appendix C.
- Pairwise Resume Comparisons: Appendix D.
- Distinguishing True Preference Distortion from Tie-Breaking Heuristics: Appendix E.
- Triplewise Color Comparisons: Appendix F.
- Triplewise Resume Comparisons: Appendix G.
- Fourwise Color Comparisons: Appendix H.
- Name Bias: Appendix I.
- Gender Bias: Appendix J.
- Additional Models: Appendix K.
- Additional Languages: Appendix L.

*Ivy College of Business, Iowa State University. haonany@iastate.edu
†Muma College of Business, University of South Florida. vardi@usf.edu
‡Paul Merage of Business School, University of California-Irvine. veecee@uci.edu

A Color Comparison Sets and Prompts

Each model was asked to perform triplewise and pairwise comparisons on four sets of three colors, categorized by tier. From best to worst, the tiers are *Ideal*, *Fair*, *Plain*, and *Harsh*. The method for generating these color sets is outlined in the Materials and Methods section of the main paper. Table S1 lists the specific colors used for GPT-4o-mini, Claude 3 Haiku, and Llama 3 8B. Color sets for additional models are reported in Appendix K.1 and Appendix L.1, respectively.

**Model**	**Tier**	**Color 1**	**Color 2**	**Color 3**
GPT-4o-mini	Ideal	Aqua Mist	Soft Sky Blue	Pale Turquoise
	Fair	Gentle Coral	Buttercream Yellow	Lilac
	Plain	Eggshell White	Antique White	Ivory
	Harsh	Onyx	Obsidian	Smoky Black
Claude 3 Haiku	Ideal	Sky Blue	Aqua	Robin's Egg Blue
	Fair	Pale Periwinkle	Pistachio	Soft Terracotta
	Plain	Pale Linen	Oyster	Fog
	Harsh	Black	Hot Pink	Brown
Llama 3 8B	Ideal	Robin's Egg Blue	Light Blue	Sky Blue
	Fair	Light Yellow	Creamy Yellow	Pale Coral
	Plain	Beige	Off-White	Taupe
	Harsh	Burgundy	Hot Pink	Charcoal Gray

Table S1: **Colors used in triplewise and pairwise comparisons.** The three colors in each row were used for triplewise comparisons for each model and tier. Colors 1 and 2 were used for pairwise comparisons.

For triplewise comparisons, each model was presented with all six permutations of the three colors and asked to select the best one for a kid's room. For pairwise comparisons, both permutations of the two colors were used.

The prompt used for pairwise comparisons was:

```
prompt = [
    f"Which color is the best for a kid's room: "
    f"{first} or {second}? Please answer with exactly "
    f"one color and no punctuation or explanation."
]
```

The prompt used for triplewise comparisons was similar and is omitted. The prompts used for Mandarin Chinese and Hebrew are reproduced in Appendix L.1.

B Resume Comparison Sets and Prompts

The selection methodology of resume for triplewise and pairwise comparisons is outlined in the Materials and Methods section of the main paper. The prompt used to generate the resumes are the following.

```
prompt = [
        f"Generate a professional resume for a highly qualified {occupation}. "
        "Ensure all details (education, work experience, achievements) are outstanding and
    appropriate for this occupation. "
        "As before, use real-world job titles, company names, and universities. Do not use
    placeholders. "
        "Put all personal information (such as name, email, phone number, address, and any
    identifying details) inside a single 'personal_information' field as a dictionary. "
        "The candidate should have exceptional experience, top-tier degrees, and major
    accomplishments. "
        "Strictly output only JSON with NO extra text or explanations. If unsure, return an empty
    JSON object: {}.",

        f"Generate a professional resume for a {occupation} that is good but clearly weaker than
    the previous one. "
        "The candidate should still be well-qualified, although clearly less qualified than the
    previous candidate and missing any top-tier credentials or major achievements. "
        "As before, use real-world job titles, company names, and universities. Do not use
    placeholders. "
        "As before, put all personal information (such as name, email, phone number, address, and
    any identifying details) inside a single 'personal_information' field as a dictionary. "
        "Strictly output only JSON with NO extra text or explanations. If unsure, return an empty
    JSON object: {}.",

        f"Generate a professional resume for a {occupation} that is noticeably weaker than the
    previous one. "
        "The candidate should have some relevant experience but fewer qualifications, weaker
    education, and minimal achievements. "
        "As before, use real-world job titles, company names, and universities. Do not use
    placeholders. "
        "As before, put all personal information (such as name, email, phone number, address, and
    any identifying details) inside a single 'personal_information' field as a dictionary. "
        "Strictly output only JSON with NO extra text or explanations. If unsure, return an empty
    JSON object: {}.",

        f"Generate a professional resume for a {occupation} that is the weakest of all. "
        "The candidate should have minimal relevant experience, lack strong qualifications, and
    have very few or no achievements. "
        "As before, use real-world job titles, company names, and universities. Do not use
    placeholders. "
        "As before, put all personal information (such as name, email, phone number, address, and
    any identifying details) inside a single 'personal_information' field as a dictionary. "
        "Strictly output only JSON with NO extra text or explanations. If unsure, return an empty
    JSON object: {}."
        ]
```

We generated synthetic personal profiles by randomly combining pre-defined lists of common U.S. Caucasian first and last names, and U.S. state abbreviations. Below are the full lists:

Last names: Smith, Johnson, Williams, Brown, Jones, Miller, Davis, Wilson, Anderson, Taylor, Thomas, Moore, Harris, Clark, Lewis.

Male first names: James, John, Robert, Michael, William, David, Joseph, Charles, Thomas, Christopher, Daniel, Matthew, Andrew, Joshua, Brandon.

Female first names: Emily, Jessica, Ashley, Sarah, Elizabeth, Hannah, Samantha, Lauren, Megan, Rachel, Amanda, Rebecca, Nicole, Stephanie, Katherine.

The male and female names were randomly permuted and matched with a last name; each last name appeared once for male candidates and once for female candidates. The prompt used for pairwise resume comparisons was:

```
prompt = [
        f"""
            You are a hiring manager evaluating two candidates for the same job. Based only on
    their resumes, select the stronger candidate.

            Candidate 1:
            {json.dumps(r1, indent=2)}

            Candidate 2:
            {json.dumps(r2, indent=2)}

            Provide only a name, without using N/A or any explanation.
        """
        ]
```

The prompt used for triplewise comparisons was similar and is omitted.

C Pairwise Color Comparisons

We used Colors 1 and 2 from Table S1 for each model and tier to conduct pairwise color comparisons. Each color set was tested in 50 pairwise comparisons for both permutations, resulting in 100 total comparisons per set at each temperature setting.

Table S2 reports the results at $T = 1$, showing the proportion of times each position was chosen, along with effect sizes (Cohen's h) and associated p-values. Effect sizes are measured against a 50% baseline, and p-values reflect the significance of deviation based on a two-sided binomial test. Even at the highest temperature setting used, most comparisons reveal statistically significant and substantial position biases. We do not perform statistical tests for $T = 0$ as the results are almost deterministic. Pairwise comparisons for additional models are reported in Appendix K.2 and Appendix L.2 respectively.

**Model**	**Tier**	**First / Second (%)**	**Cohen's *h***	***p*-value**
GPT-4o-mini	Ideal	98 / 2	1.29	$\mathbf{8.0 \times 10^{-27}}$***
	Fair	89 / 11	0.90	$\mathbf{2.5 \times 10^{-16}}$***
	Plain	31 / 69	0.39	$\mathbf{1.8 \times 10^{-4}}$***
	Harsh	21 / 79	0.62	$\mathbf{4.3 \times 10^{-9}}$***
Claude 3 Haiku	Ideal	73 / 27	0.48	$\mathbf{4.7 \times 10^{-6}}$***
	Fair	15 / 85	0.78	$\mathbf{4.8 \times 10^{-13}}$***
	Plain	20 / 80	0.64	$\mathbf{1.1 \times 10^{-9}}$***
	Harsh	15 / 85	0.78	$\mathbf{4.8 \times 10^{-13}}$***
Llama 3 8B	Ideal	72 / 28	0.46	$\mathbf{1.3 \times 10^{-5}}$***
	Fair	86 / 14	0.80	$\mathbf{8.3 \times 10^{-14}}$***
	Plain	60 / 40	0.20	0.057
	Harsh	16 / 84	0.75	$\mathbf{2.6 \times 10^{-12}}$***

Table S2: **Position bias in pairwise color comparisons, $T = 1$.** Proportions of first and second selections across quality tiers. Cohen's h quantifies effect sizes from a 50% baseline. Asterisks indicate significance: $\mathbf{p < 0.001}$***.

Figure S1 shows the results at different temperatures ($T = 0, 0.5$, and 1). At $T = 0$ (the topmost plot), all models strongly prefer the first option for high-quality color sets, and shift to the second position for lower-quality sets. For instance, GPT-4o-mini displayed a primacy effect in the top two tiers (Ideal and Fair) and a recency effect in the bottom two (Plain and Harsh). Claude 3 Haiku and Llama 3 8B showed a primacy effect in the top one and three tiers, respectively, and a recency effect in the bottom three and one tiers, respectively. While higher temperatures introduce randomness, the position bias remains robust across models and tiers.

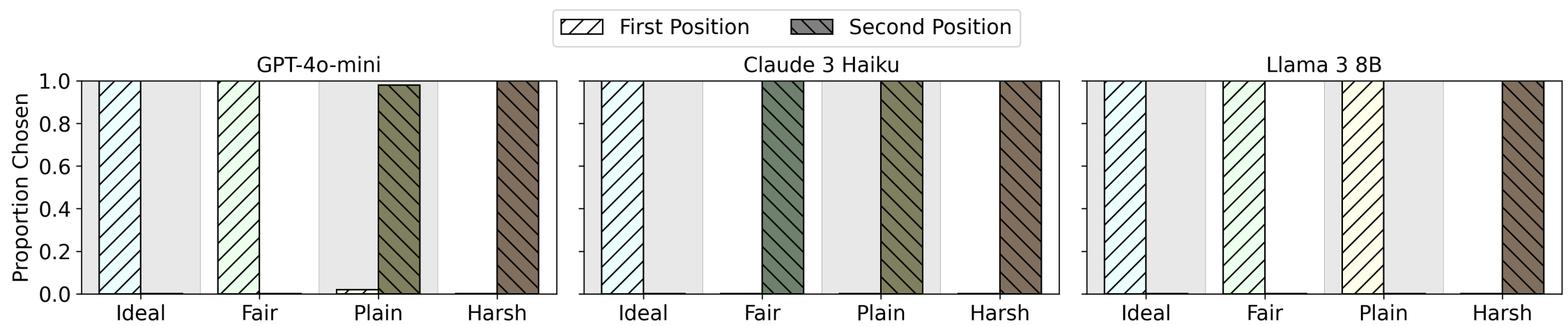

(a) Comparisons at temperature 0

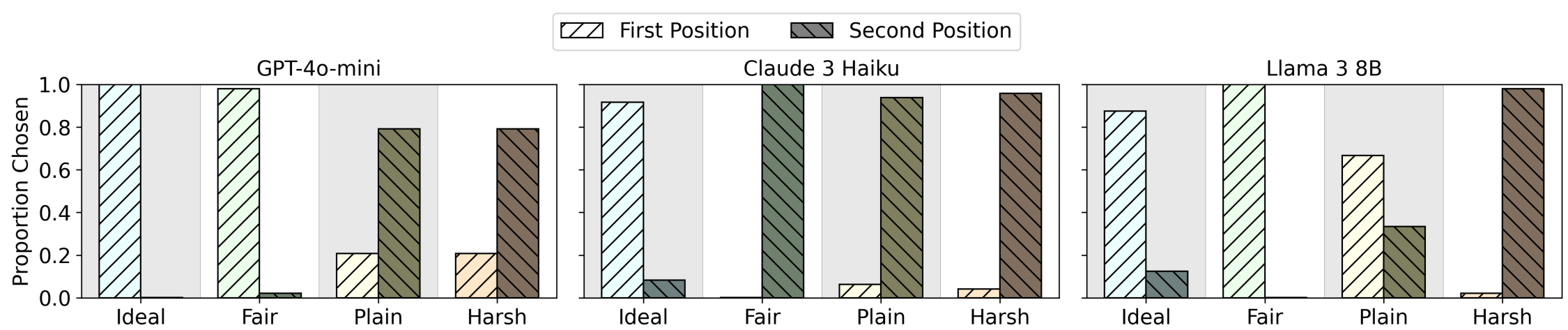

(b) Comparisons at temperature 0.5

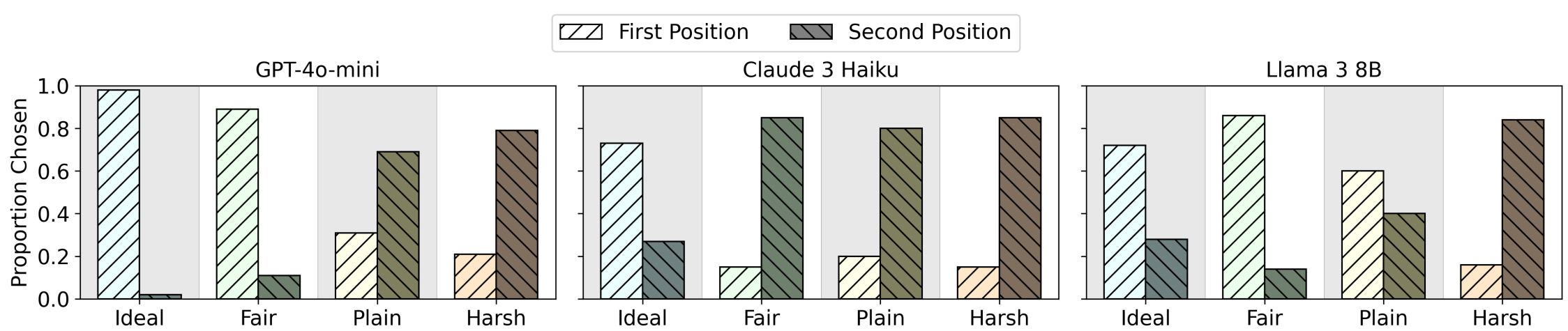

(c) Comparisons at temperature 1

Figure S1: **Order effects in pairwise comparisons at three temperature settings.** Each panel ($T = 0, 0.5$, and 1, top to bottom) shows the percentage of selections by position, broken down by model and color tier.

D Pairwise Resume Comparisons

For pairwise resume comparisons, we selected two resumes as outlined in the Materials and Methods section. We randomly selected four male names and four female names, and conducted exhaustive pairwise comparisons: 2 genders $\times$ $\binom{4}{2}$ name pairs $\times$ 2 name-resume assignments $\times$ 2 presentation orders, for a total of 48 comparisons per tier and profession.

The names used for pairwise comparisons were *Brandon Lewis, Charles Wilson, Christopher Taylor* and *Andrew Harris* (male) and *Elizabeth Jones, Ashley Williams, Amanda Thomas* and *Emily Smith* (female).

Table S3 shows the proportion of times each position was chosen at $T = 1$, along with effect sizes (Cohen's h) and associated p-values. As in Appendix C, effect sizes are measured against a 50% baseline, and p-values reflect the significance of deviation based on a two-sided binomial test.

Figure S2 and Figure S3 plot the positional effect results for the four professions, as well as aggregated results, at temperatures 0 and 1 respectively. The results align with findings from the color experiment: all models tend to prefer the first position for high-quality resumes but shift to favor the second position for low-quality ones. Figure S3 shows the corresponding results at $T = 1$. Results for temperature 0.5 are consistent with the other findings and are omitted.

Note that there is a difference between the pairwise color results at $T = 0$ (Figure S1a) and the pairwise resume results (Figure S2). Specifically, in the color results, each color is chosen almost exactly 50% of the time. This matches the expectation that $\Pr[a \mid \{a, b\}, 0]$ will be very close to 0, 0.5, or 1.[1]

At first glance, some pairwise resume comparisons seem to deviate from this pattern. The reason is that our resume dataset combines the same core content with different personal information. For example, let resumes X and Y be the core contents for the two resumes in the *Good* tier for Claude 3 Haiku, and let BL and CW be two male names. XBL denotes resume X with the personal information of BL.

Because of the name bias, comparing XBL with YCW may yield different outcomes than comparing XCW with YBL. For example, if

$$\Pr[\text{X}_{\text{BL}} \mid \{\text{X}_{\text{BL}}, \text{Y}_{\text{CW}}\}, 0] = 1 \quad \text{and} \quad \Pr[\text{X}_{\text{CW}} \mid \{\text{X}_{\text{CW}}, \text{Y}_{\text{BL}}\}, 0] = 0.5,$$

then core content X will be selected 75% of the time when results are aggregated over these two names. Since we use two core resumes and four names for each gender, this yields 12 $\{a, b\}$ pairs per gender, allowing a wide range of aggregate probabilities to emerge. Thus, deviations from exactly 0%, 50%, or 100% in the resume results are explained by the interaction between name bias and aggregation across name–content combinations. For more on the name bias, see Section I.

[1] Recall that $\Pr[a \mid \{a, b\}, t]$ is the overall probability of selecting a when both permutations (a, b) and (b, a) are presented an equal number of times at temperature t.

Model	Tier	First / Second (%)	Cohen's h	p-value
GPT-4o-mini	Best	100 / 0	1.57	$\mathbf{3.2 \times 10^{-58}}^{***}$
	Good	23 / 77	0.57	$\mathbf{2.4 \times 10^{-14}}^{***}$
	Mediocre	3 / 97	1.25	$\mathbf{6.8 \times 10^{-49}}^{***}$
	Weak	0 / 100	1.57	$\mathbf{3.2 \times 10^{-58}}^{***}$
Claude 3 Haiku	Best	63 / 37	0.25	$\mathbf{6.6 \times 10^{-4}}^{***}$
	Good	11 / 89	0.88	$\mathbf{1.6 \times 10^{-29}}^{***}$
	Mediocre	1 / 99	1.43	$\mathbf{6.2 \times 10^{-56}}^{***}$
	Weak	0 / 100	1.57	$\mathbf{3.2 \times 10^{-58}}^{***}$
Llama 3 8B	Best	82 / 18	0.70	$\mathbf{2.6 \times 10^{-20}}^{***}$
	Good	83 / 17	0.72	$\mathbf{5.6 \times 10^{-21}}^{***}$
	Mediocre	8 / 92	0.99	$\mathbf{3.0 \times 10^{-35}}^{***}$
	Weak	0 / 100	1.57	$\mathbf{3.2 \times 10^{-58}}^{***}$

Table S3: **Pairwise position bias in resume comparisons, $T = 1$.** Results are aggregated across all professions, grouped by model and tier. For each condition, we report the proportion of times the first vs. second resume was chosen (First/Second %), the corresponding Cohen's h effect size, and the p-value from a two-sided binomial test against a 50% null. All p-values are from binomial test of uniform choice; $\mathbf{p < 0.001}^{***}$ indicates statistical significance.

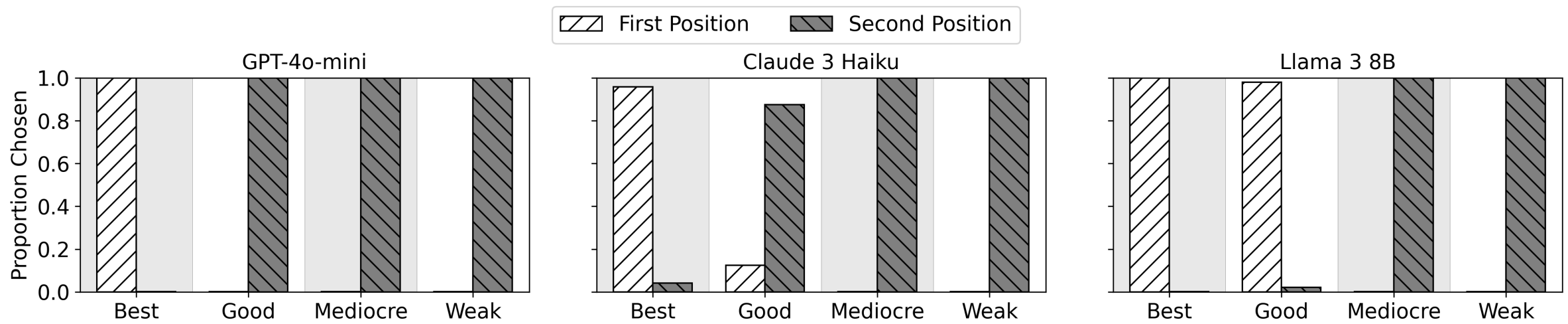

(a) Mechanical Engineer

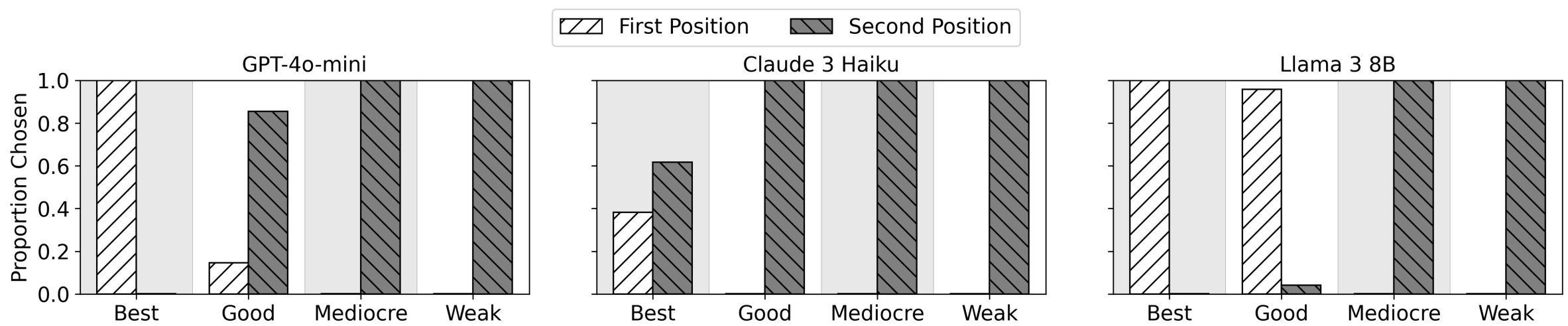

(b) Real Estate Agent

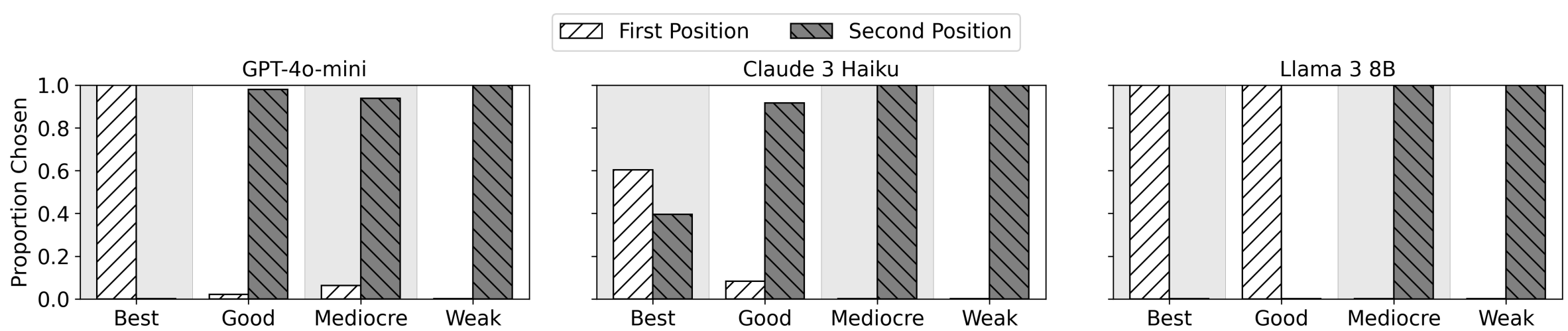

(c) Journalist

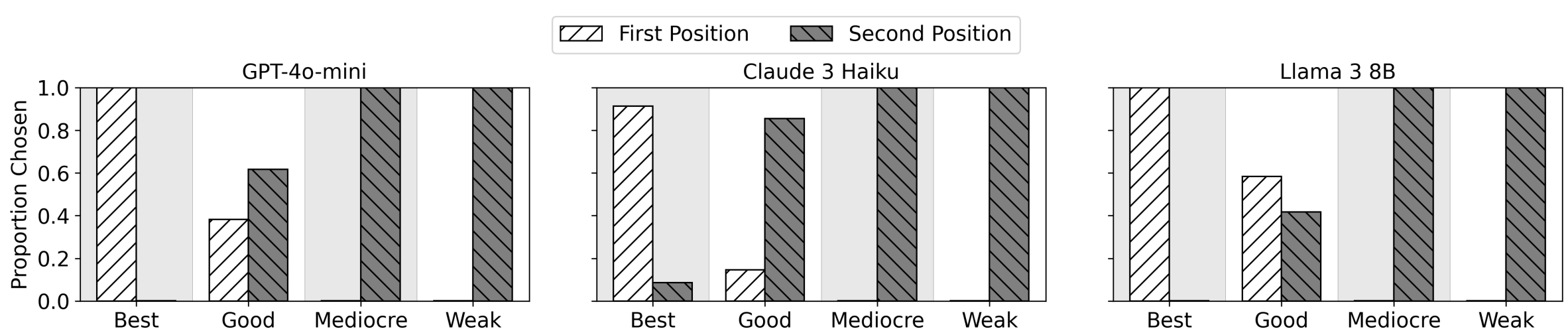

(d) Registered Nurse

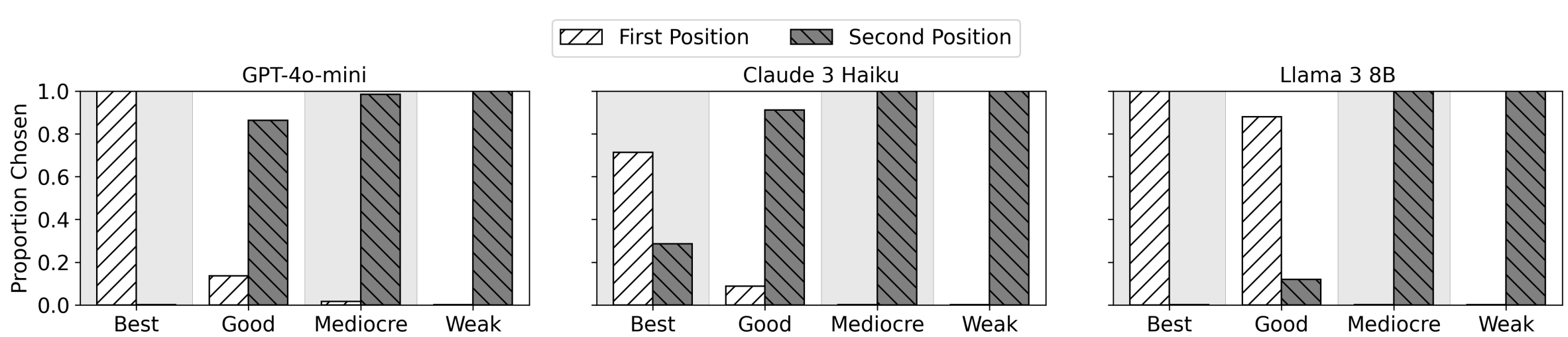

(e) Aggregated across all four professions

Figure S2: **Order effects in pairwise resume comparisons, $T = 0$.** Each panel shows the proportion of times each position was chosen for each profession (Mechanical Engineer, Real Estate Agent, Journalist and Registered Nurse, top to bottom) and aggregated, broken down by LLM and quality tier.

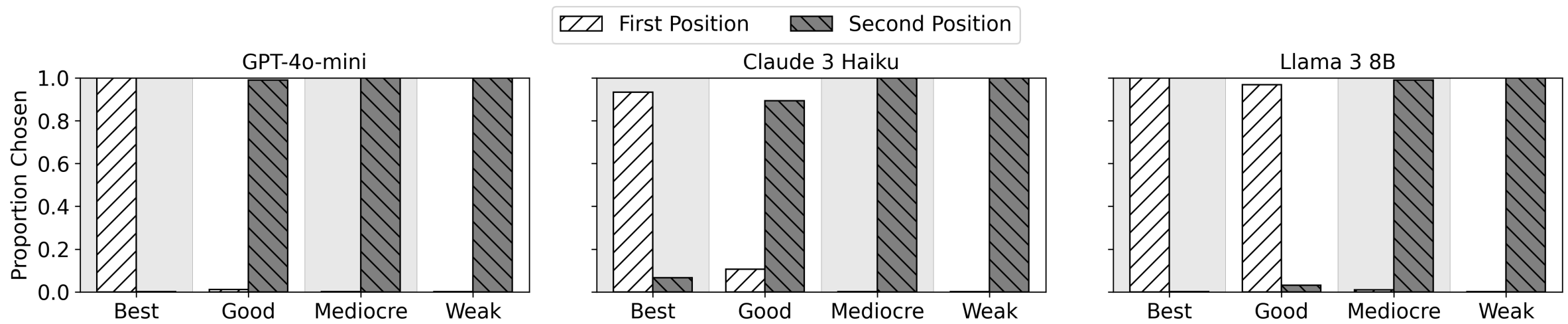

(a) Mechanical Engineer

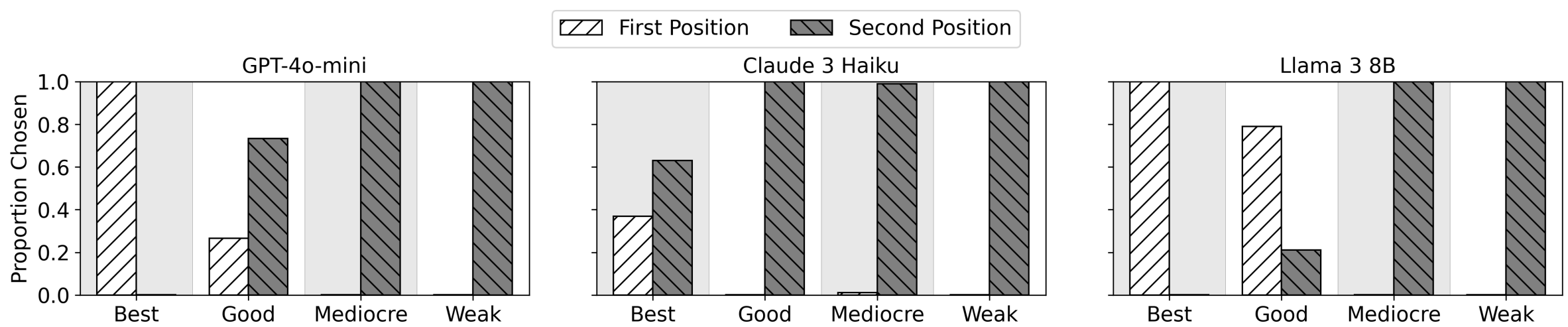

(b) Real Estate Agent

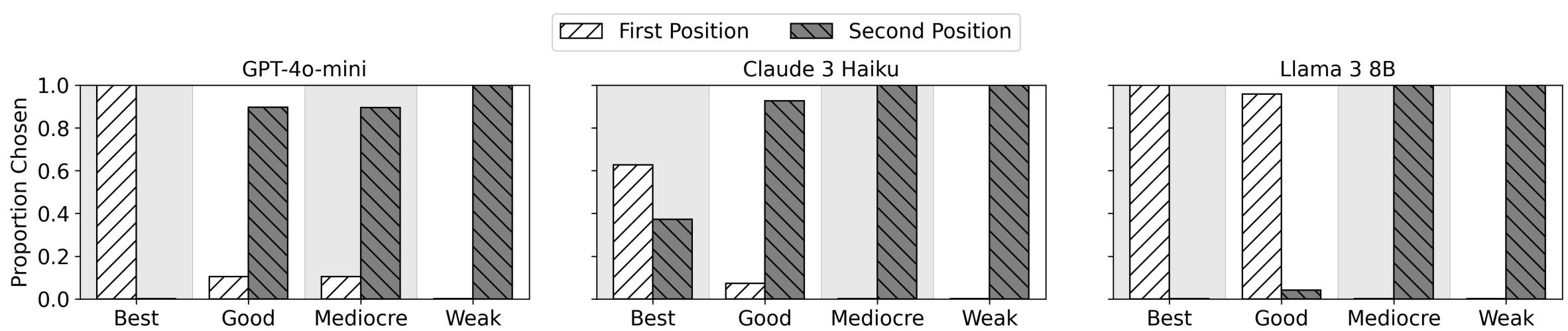

(c) Journalist

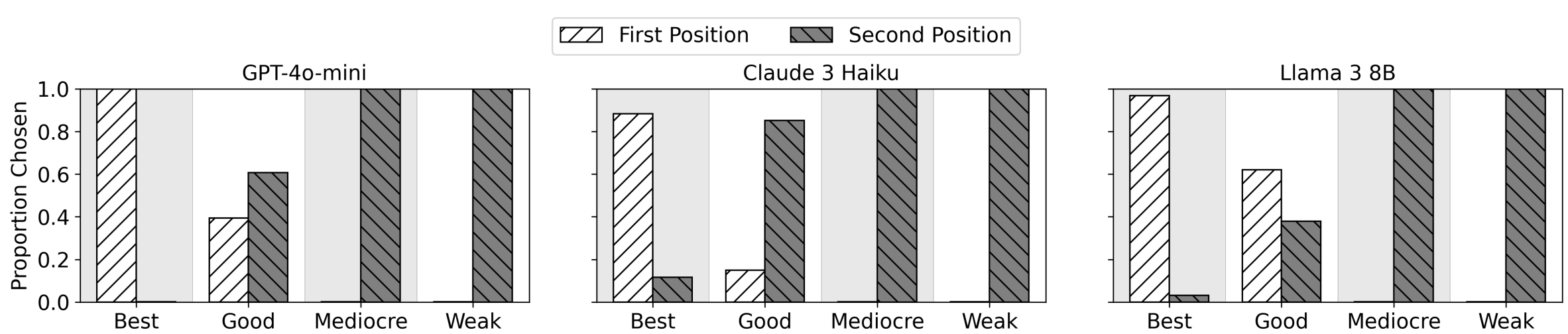

(d) Registered Nurse

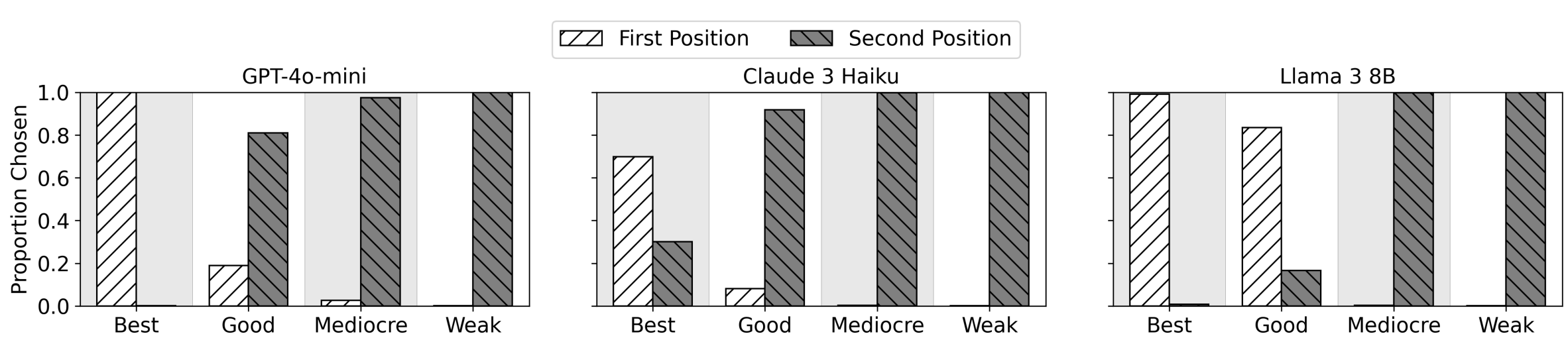

(e) Aggregated across all four professions

Figure S3: **Order effects in pairwise resume comparisons, $T = 1$.** Each panel shows the proportion of times each position was chosen for each profession (Mechanical Engineer, Real Estate Agent, Journalist, and Registered Nurse, top to bottom) and aggregated, broken down by LLM and quality tier.

E Distinguishing True Preference Distortions from Tie-Breaking Heuristics.

As reported in Appendix C, we conducted 50 comparisons for each permutation of each pair of colors at each temperature. Here, we conducted an additional 500 pairwise tests (250 iterations per permutation), for a total of 600 pairwise comparisons. The results are shown in Table S4. In eight of the twelve tests, the model's preference was statistically significant. Even when there is a clear preference, the effect size is small. This is to be expected, as the colors were chosen so that the LLM would be indifferent between them at $T = 0$.

In order to try to minimize the impact of name and gender biases observed in pairwise resume comparisons, we selected two names that we found evoked minimal name biases when compared to each other: Ashley Williams and Emily Smith. Similarly to the color selection, we conducted 600 pairwise comparisons for resumes. Here, we needed to compare Resume 1 with Ashley Williams' personal information to Resume 2 with Emily Smith's personal information and vice versa. We therefore performed 150 iterations per permutation and resume-name pair, for a total of 600 pairwise comparisons. Here, the model's preference was statistically significant in five of the twelve resume pairs. The results for resume comparisons are shown in Table S5.

**Model**	**Tier**	**C1 / C2 (%)**	**Cohen's h**	**p-value**
GPT-4o-mini	Ideal	53 / 47	0.05	0.118
	Fair	56 / 44	0.11	$\mathbf{3.1 \times 10^{-3}}^{**}$
	Plain	55 / 45	0.09	$\mathbf{0.012}^{*}$
	Harsh	56 / 44	0.11	$\mathbf{3.1 \times 10^{-3}}^{**}$
Claude 3 Haiku	Ideal	46 / 54	0.08	$\mathbf{0.023}^{*}$
	Fair	49 / 51	0.03	0.244
	Plain	64 / 36	0.28	$\mathbf{2.0 \times 10^{-11}}^{***}$
	Harsh	61 / 39	0.22	$\mathbf{1.1 \times 10^{-7}}^{***}$
Llama 3 8B	Ideal	47 / 53	0.05	0.118
	Fair	46 / 54	0.07	$\mathbf{0.039}^{*}$
	Plain	52 / 48	0.04	0.184
	Harsh	35 / 65	0.31	$\mathbf{3.9 \times 10^{-14}}^{***}$

Table S4: **Distinguishing tie-breaking from true distortions (colors).** Values in the "C1/C2 (%)" column indicate the percentage of times each color was chosen the first color, where C1 and C2 denote the first and second color of that tier, denoted 'Color 1' and 'Color 2' in Table S1, respectively. Cohen's h quantifies effect size. Asterisks denote significance: $^{*}p < .05$, $^{**}p < .01$, $^{***}p < .001$.

Model	Tier	R1 / R2 (%)	Cohen’s h	p-value
GPT-4o-mini	Best	50 / 50	0.00	1.00
	Good	57 / 43	0.28	$\mathbf{9.3 \times 10^{-4}}$***
	Mediocre	43 / 57	0.28	$\mathbf{6.9 \times 10^{-4}}$***
	Weak	51 / 49	0.05	0.60
Claude 3 Haiku	Best	49 / 51	0.03	0.78
	Good	50 / 50	0.00	0.90
	Mediocre	50 / 50	0.00	1.00
	Weak	50 / 50	0.00	1.00
Llama 3 8B	Best	57 / 43	0.28	$\mathbf{5.1 \times 10^{-4}}$***
	Good	48 / 52	0.07	0.39
	Mediocre	66 / 34	0.66	$\mathbf{1.9 \times 10^{-15}}$***
	Weak	54 / 46	0.17	**0.045***

Table S5: **Distinguishing tie-breaking from true distortions (resumes).** Values in the “R1/R2 (%)” column indicate the percentage of times each resume was chosen, where R1 and R2 denote the two resumes selected for pairwise comparisons. Cohen’s h quantifies effect size. Asterisks denote significance: $^{*}p < .05$, $^{**}p < .01$, $^{***}p < .001$.

F Triplewise Color Comparisons

For triplewise comparisons, we used all three colors from Table S1 in Appendix A. Each of the $3! = 6$ permutations was iterated 40 times, for a total of 240 triplewise comparisons.

We used a chi-square test to assess whether LLMs exhibit position bias when evaluating three colors of (approximately) equal quality. Under the null hypothesis of no positional preference, we would expect the model to select each position with equal probability (i.e., one-third each). Note that this is regardless of quality differentials: even if one color is significantly preferred to the others, we would expect each position to be selected one-third of the time. A statistically significant deviation from this uniform distribution (as indicated by $p < 0.001$) suggests that the model's selection is systematically influenced by resume order rather than content. To quantify the strength of this effect, we report Cramér's V as a measure of effect size. Table S6 shows the results of the triplewise comparisons at $T = 1$, showing the proportion of times each position was chosen, along with effect sizes and associated p-values.

Figure S4 shows the results at different temperatures. Similarly to the pairwise comparisons, while higher temperatures introduce randomness, the position bias remains robust.

**Model**	**Tier**	**First/Second/Third (%)**	**Cramér's V**	**p-value**
GPT-4o-mini	Ideal	81 / 16 / 3	0.73	$\mathbf{7.1 \times 10^{-56}}$***
	Fair	55 / 31 / 14	0.36	$\mathbf{2.0 \times 10^{-14}}$***
	Plain	3 / 83 / 14	0.75	$\mathbf{3.1 \times 10^{-59}}$***
	Harsh	5 / 31 / 64	0.51	$\mathbf{8.3 \times 10^{-28}}$***
Claude 3 Haiku	Ideal	61 / 32 / 7	0.46	$\mathbf{4.4 \times 10^{-23}}$***
	Fair	0 / 20 / 80	0.72	$\mathbf{6.0 \times 10^{-55}}$***
	Plain	4 / 28 / 68	0.56	$\mathbf{2.2 \times 10^{-33}}$***
	Harsh	14 / 69 / 17	0.53	$\mathbf{3.3 \times 10^{-30}}$***
Llama 3 8B	Ideal	60 / 16 / 24	0.40	$\mathbf{1.4 \times 10^{-17}}$***
	Fair	40 / 10 / 49	0.35	$\mathbf{1.2 \times 10^{-13}}$***
	Plain	21 / 24 / 55	0.33	$\mathbf{8.8 \times 10^{-12}}$***
	Harsh	5 / 15 / 80	0.71	$\mathbf{1.0 \times 10^{-52}}$***

Table S6: **Triplewise color comparisons, $T = 1$.** Proportions chosen from each position across models and quality tiers, reported as First/Second/Third (%). All p-values from chi-square tests of uniform choice; $\mathbf{p < 0.001}$*** denotes statistical significance.

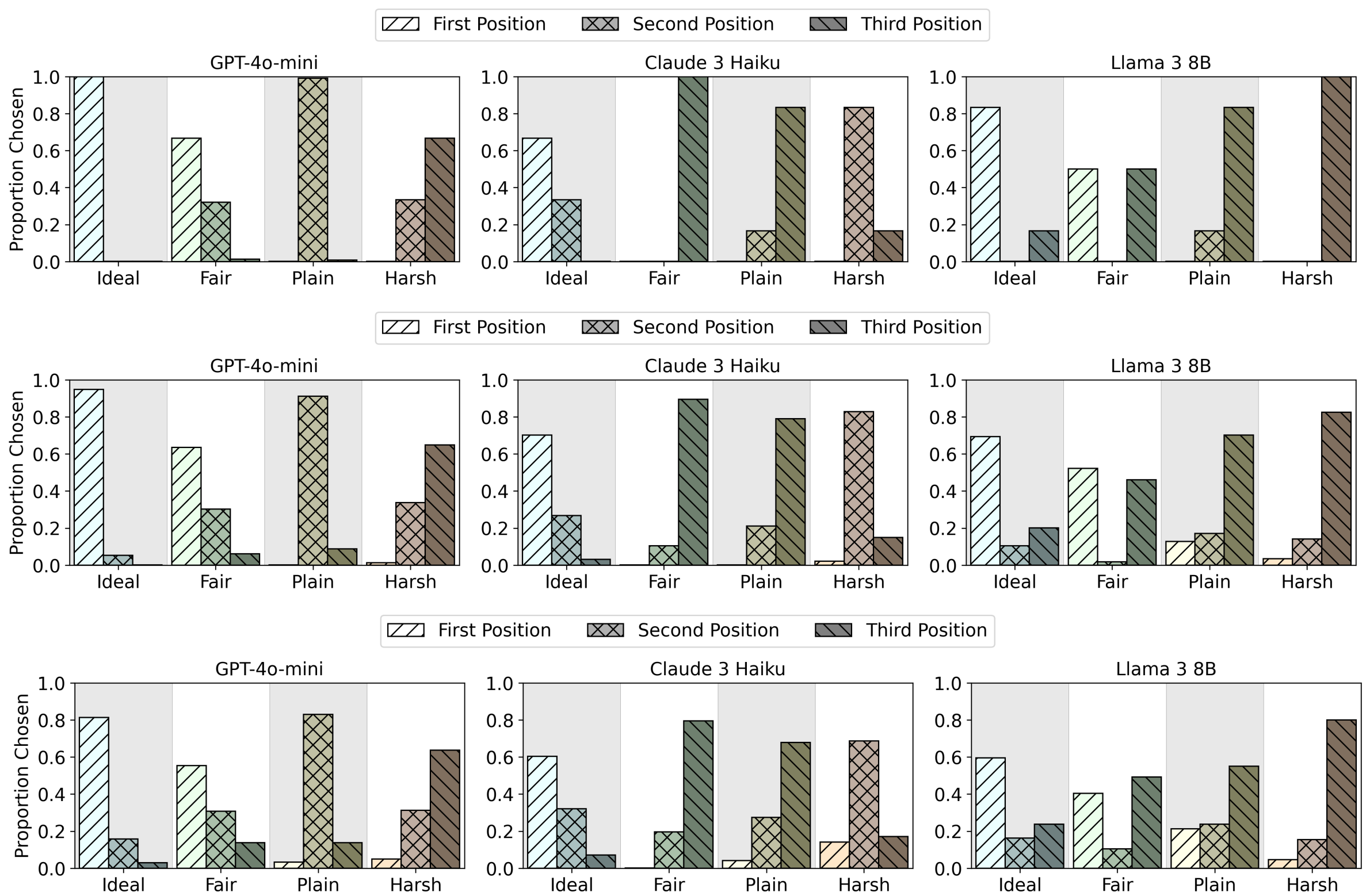

Figure S4: **Order effects in triplewise comparisons at three temperature settings.** Each panel ($T = 0, 0.5$, and 1, top to bottom) shows the percentage of selections by position, broken down by model and color tier.

G Triplewise Resume Comparisons

The method for generating the resumes for triplewise comparisons is outlined in the Materials and Methods section. Similarly to the triplewise color comparisons, we conducted a chi-squared goodness-of-fit test under the null hypothesis that each position is selected with equal probability (i.e., one third for each of the three positions), and measured effect sizes using Cramér's V. Table S7 shows the results of the triplewise comparisons at $T = 1$, showing the proportion of times each position was chosen, along with effect sizes and associated p-values.

Figure S5 and Figure S6 visualize the position effect results for the four professions, as well as aggregated results, at temperatures 0 and 1 respectively. Triplewise comparisons for additional models are reported in Appendix K.3 and Appendix L.3 respectively.

**Model**	**Tier**	**First / Second / Third (%)**	$\boldsymbol{\chi^2}$	**Cramér's $\boldsymbol{V}$**	$\boldsymbol{p}$**-value**
GPT-4o-mini	Ideal	90 / 9 / 1	90.62	0.86	$\mathbf{4.0 \times 10^{-13}}^{***}$
	Fair	8 / 86 / 6	77.85	0.80	$\mathbf{3.6 \times 10^{-12}}^{***}$
	Plain	2 / 92 / 6	93.55	0.88	$\mathbf{5.3 \times 10^{-16}}^{***}$
	Harsh	3 / 82 / 15	72.05	0.76	$\mathbf{1.3 \times 10^{-9}}^{***}$
Claude 3 Haiku	Ideal	52 / 20 / 28	19.98	0.36	0.180
	Fair	29 / 47 / 24	9.38	0.27	0.058
	Plain	6 / 36 / 58	32.05	0.51	$\mathbf{1.9 \times 10^{-4}}^{***}$
	Harsh	2 / 24 / 74	51.15	0.65	$\mathbf{2.0 \times 10^{-8}}^{***}$
Llama 3 8B	Ideal	61 / 16 / 23	34.75	0.49	0.053
	Fair	48 / 15 / 37	13.28	0.32	0.088
	Plain	9 / 38 / 52	21.93	0.40	$\mathbf{0.026}^{*}$
	Harsh	3 / 35 / 62	33.48	0.52	$\mathbf{1.2 \times 10^{-5}}^{***}$

Table S7: **Order bias in triplewise resume comparisons, $\boldsymbol{T = 1}$.** For each model and quality tier, "First/Second/Third (%)" shows the percentage of times the first, second, or third option was chosen. Position bias was evaluated with a chi-square goodness-of-fit test (χ^2) against uniform choice; Cramér's V is the effect size. Asterisks denote $^{*}p < 0.05$, $^{**}p < 0.01$, $^{***}p < 0.001$.

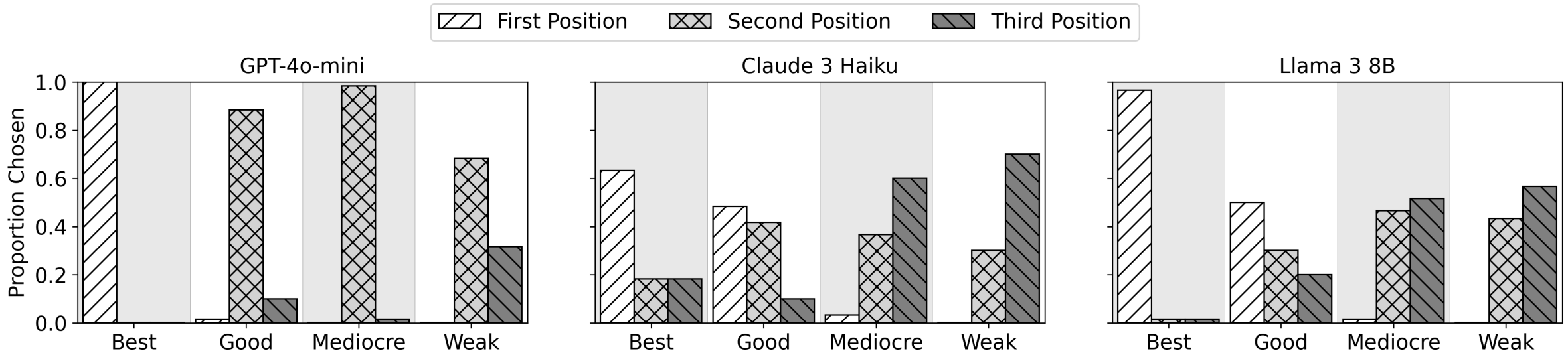

(a) Mechanical Engineer

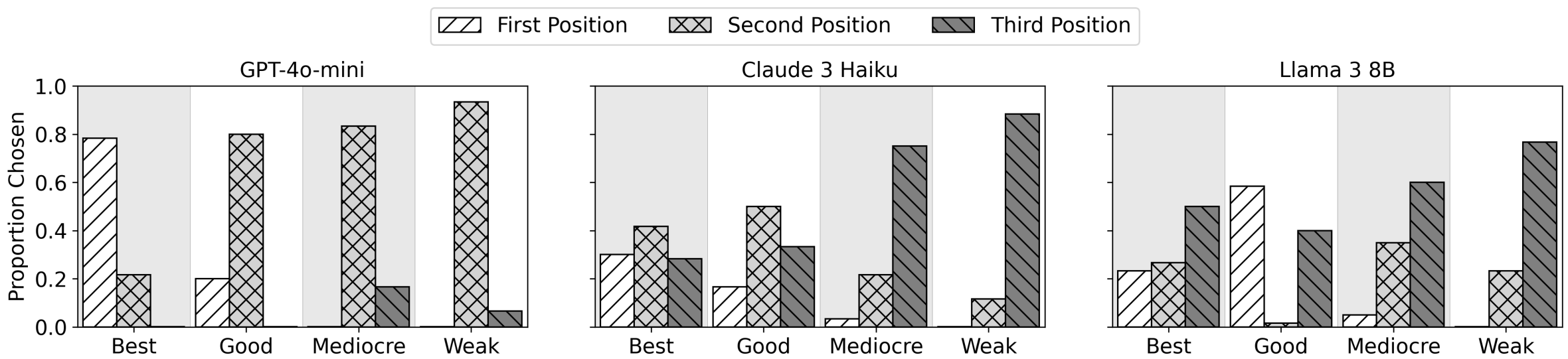

(b) Real Estate Agent

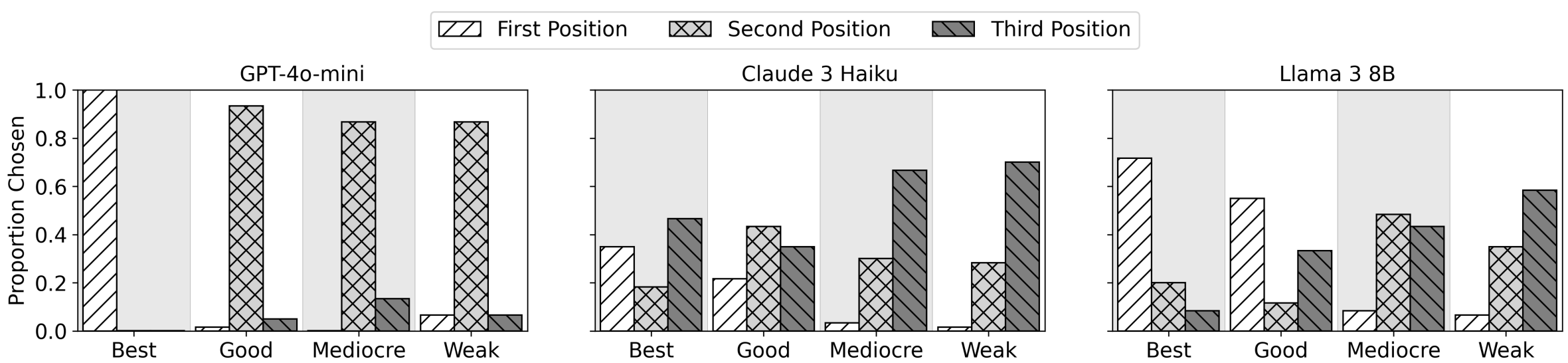

(c) Journalist

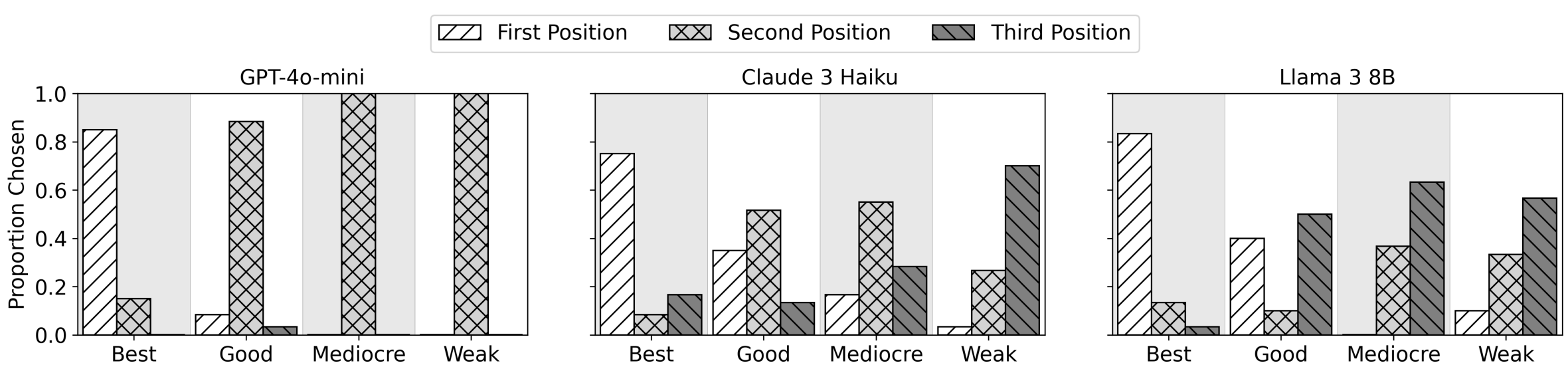

(d) Registered Nurse

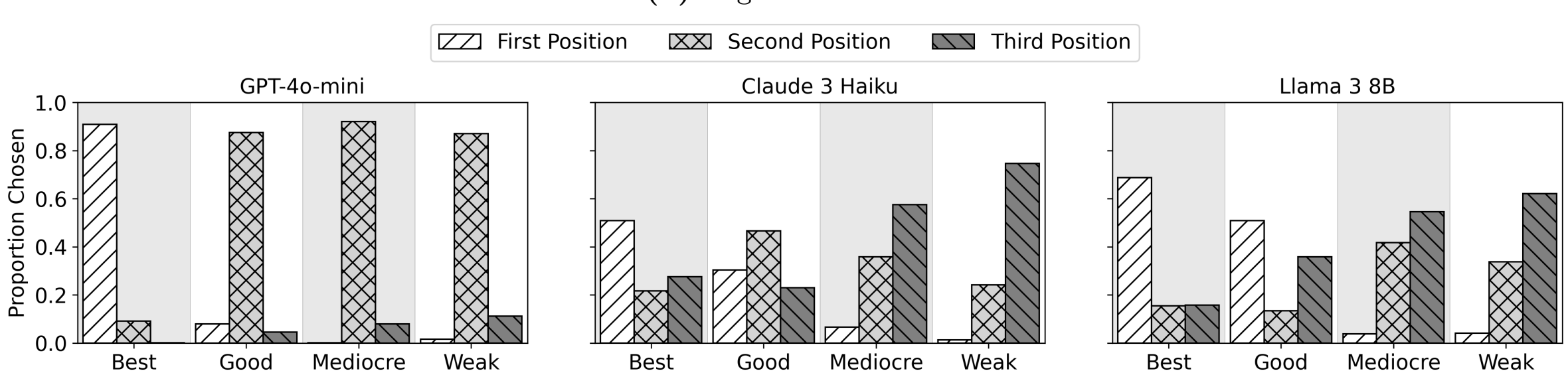

(e) Aggregated across all four professions

Figure S5: **Position Bias of triplewise resume comparisons, $T = 0$**. Positional order effects in triplewise comparisons across four professions (Mechanical Engineer, Real Estate Agent, Journalist and Registered Nurse, top to bottom) and aggregated across professions.

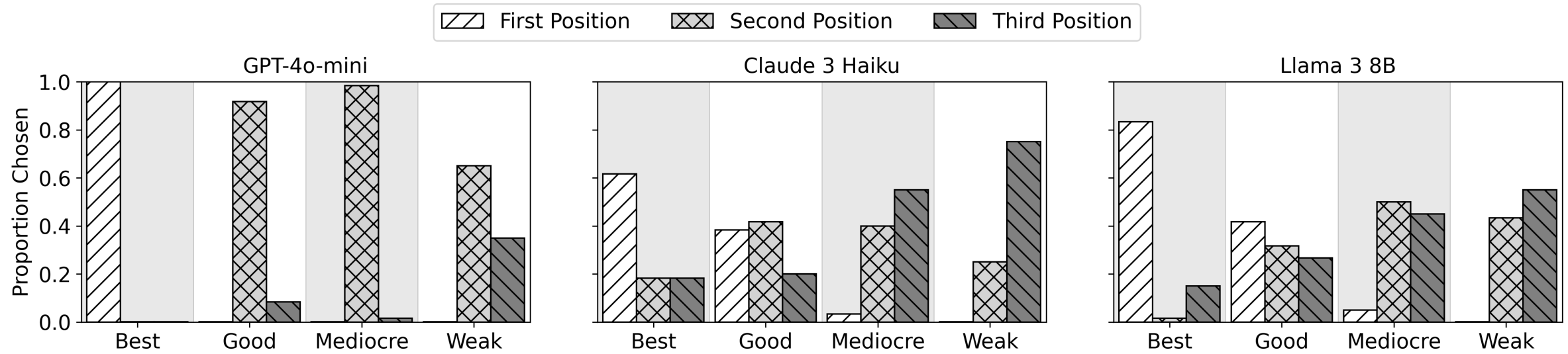

(a) Mechanical Engineer

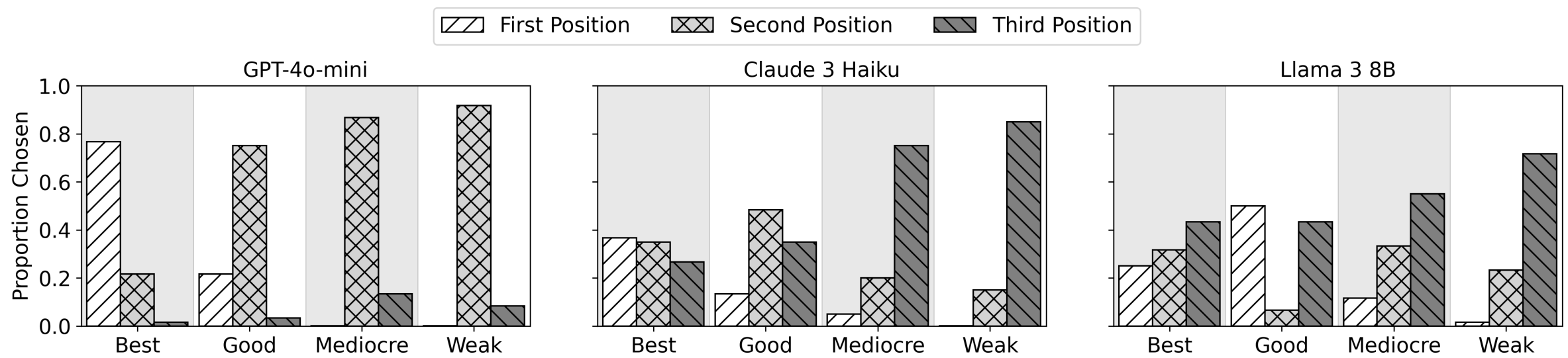

(b) Real Estate Agent

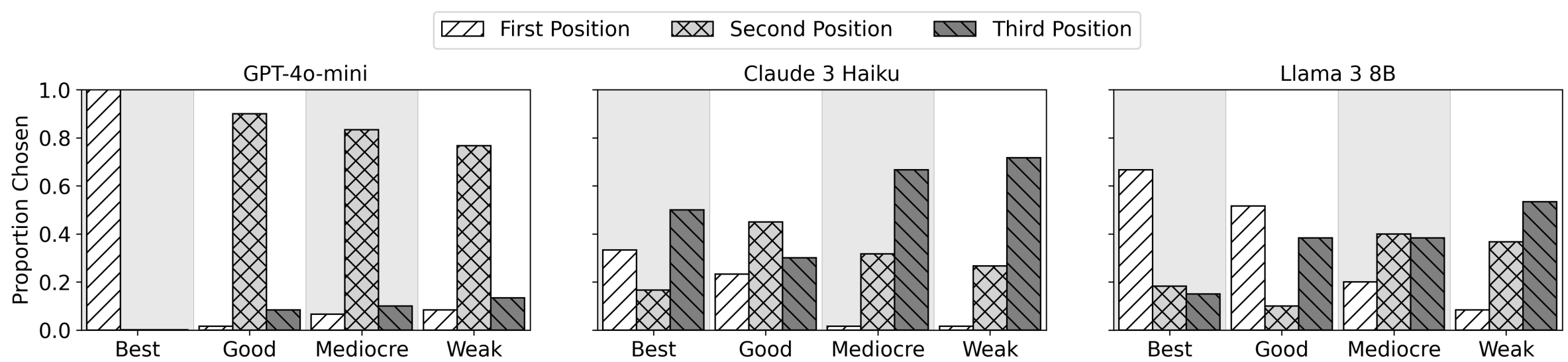

(c) Journalist

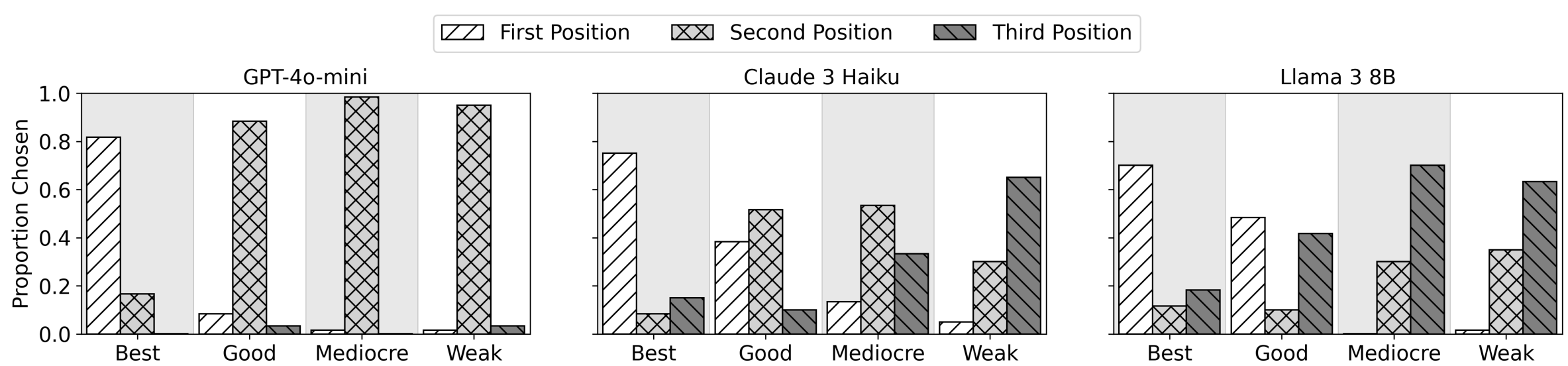

(d) Registered Nurse

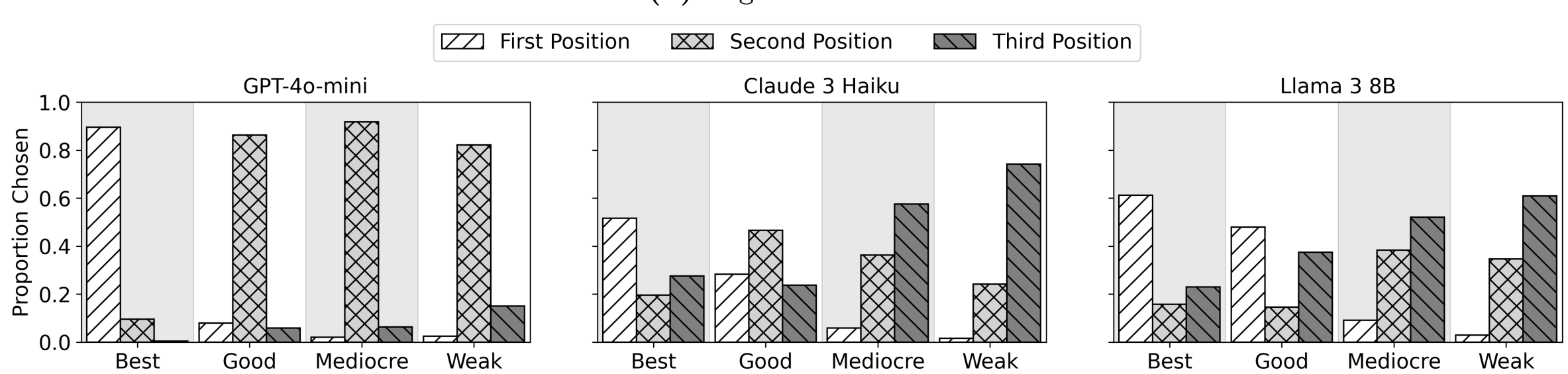

(e) Aggregated across all four professions

Figure S6: **Triplewise comparisons, $T = 1$.** Positional order effects in triplewise comparisons across four professions (Mechanical Engineer, Real Estate Agent, Journalist and Registered Nurse, top to bottom) and aggregated totals.

H Fourwise Color Comparisons

For fourwise comparisons, we manually curated a set of four colors, chosen to be roughly comparable, for each LLM. For each model, we presented all $4! = 24$ permutations of these colors. We repeated each permutation four times at temperature 0, yielding 96 total queries per model.

The colors selected were:

- **GPT-4o-mini:** Aqua Mist, Robin’ s Egg Blue, Soft Sky Blue, Pale Turquoise,
- **Claude 3 Haiku:** Pale Periwinkle, Pistachio, Blush Pink, Soft Terracotta,
- **Llama 3 8B:** Emerald Green, Taupe, Navy Blue, Beige.

For GPT-4o-mini, the color set included its three colors from the *Ideal* tier, with Robin’ s Egg Blue added. For Claude 3 Haiku, the colors came from its *Fair* tier, with Pistachio added. For Llama 3 8B, two colors were drawn from its *Plain* tier, with Emerald Green and Navy Blue added. Selection frequencies among the different colors were less balanced than in pairwise and triplewise comparisons, but each color was chosen at least once per repetition. GPT-4o-mini selected Aqua Mist and Robin’ s Egg Blue 30 times each, Soft Sky Blue 24 times, and Pale Turquoise 12 times. Claude 3 Haiku most often selected Pale Periwinkle (42), followed by Pistachio (26), Blush Pink (24), and Soft Terracotta (4). Llama 3 8B favored Emerald Green (40), then Taupe (28), Navy Blue (20), and Beige (8).

Figure S7 shows the proportion of times each position (first, second, third, fourth) was chosen across all permutations, indicating consistent positional preferences in fourwise comparisons across all three models.

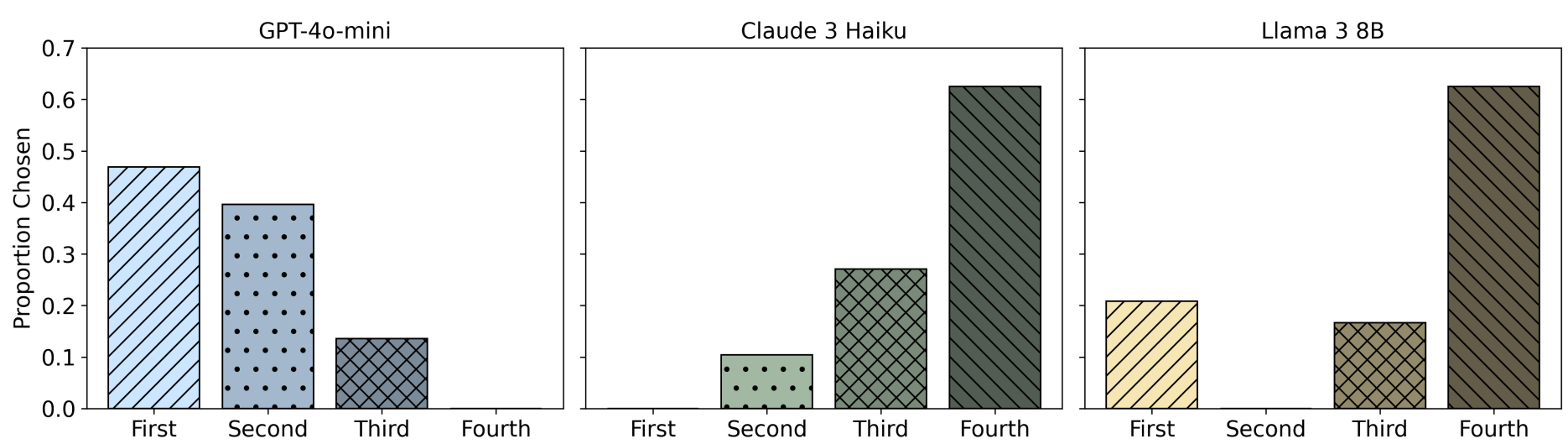

Figure S7: **Fourwise position bias across models, $T = 0$.** Proportion of times each position was chosen in four-option comparisons. Bar colors progressively darken to indicate position rank. All models show substantial positional preferences, suggesting robust order-driven distortions in higher-choice settings.

I Name Bias

I.1 Name Bias in Triplewise Comparisons

To evaluate whether name selections were uniformly distributed across the 30 candidate names for each model, we performed a chi-squared goodness-of-fit test. The results are shown in Table S8: GPT-4o-mini's selections closely match a uniform distribution ($\chi^2 = 9.6$, $p = 0.9997$), while Claude 3 Haiku shows a strong deviation ($\chi^2 = 215.8$, $p = 1.9 \times 10^{-30}$), indicating a strong name-level selection bias. Llama falls between these extremes ($\chi^2 = 73.1$, $p = 1.1 \times 10^{-5}$).

Figure S8 visualizes the full distribution of name selection frequencies for each model using histograms with bin size 3. The expected number of times each name would be chosen if there was no name bias is 32. GPT-4o-mini chose each name between 26 and 38 times, consistent with little to no bias. Claude 3 Haiku 's selections show high variance, with a wide spread and strong preference for certain names. Figure S9 shows box plots for each model, confirming these patterns: Claude 3 Haiku has the widest interquartile range and largest spread, while GPT-4o-mini's selection counts are narrowly distributed with minimal outliers. Table S9 shows Claude 3 Haiku's the top 5 least and most 'favorite' names: it chose some names as few as 8 times, and others as much as 62 times.

**Model**	**Gender**	$\boldsymbol{\chi^2}$	$\boldsymbol{p}$**-value**	$\boldsymbol{n}$
GPT-4o-mini	Male	4.50	0.99	480
	Female	5.12	0.98	480
	All	9.62	1.00	960
Claude 3 Haiku	Male	100.9	$\mathbf{3.2 \times 10^{-15}}$***	480
	Female	114.9	$\mathbf{6.3 \times 10^{-18}}$***	480
	All	215.8	$\mathbf{1.9 \times 10^{-30}}$***	960
Llama 3 8B	Male	19.62	0.142	480
	Female	53.44	$\mathbf{1.6 \times 10^{-6}}$***	480
	All	73.06	$\mathbf{1.1 \times 10^{-5}}$***	960

Table S8: **Name bias in triplewise comparisons, $\boldsymbol{T = 1}$.** For each model, we report separate goodness-of-fit χ^2 statistics and p-values for male names, female names, and combined (All), along with the total number of comparisons (n). Asterisks indicate statistical significance at $^{***}p < .001$.

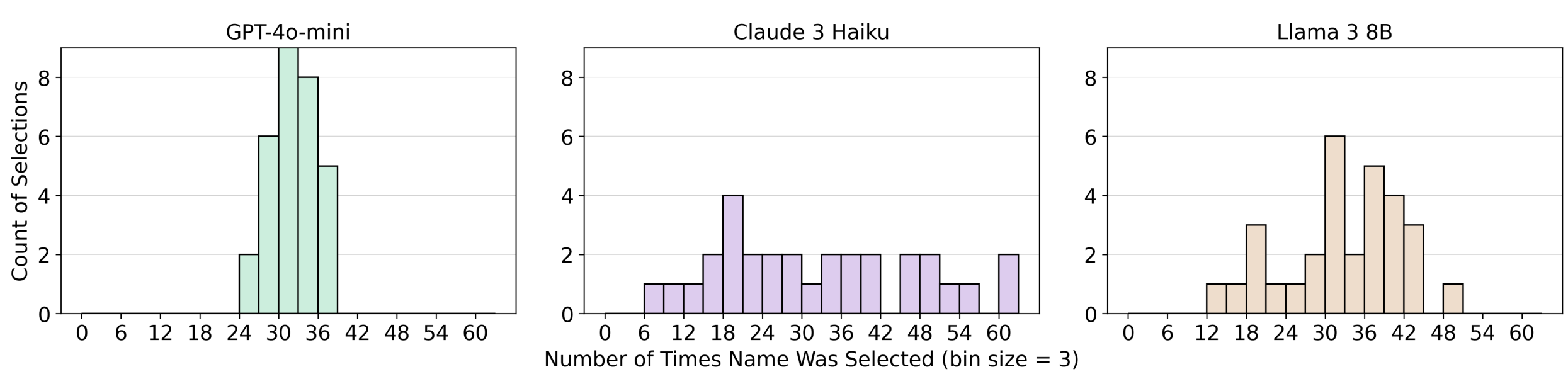

Figure S8: **Histogram of name selections per model**, binned every 3. The histogram shows how often each of the 30 candidate names was chosen across all triplewise comparisons.

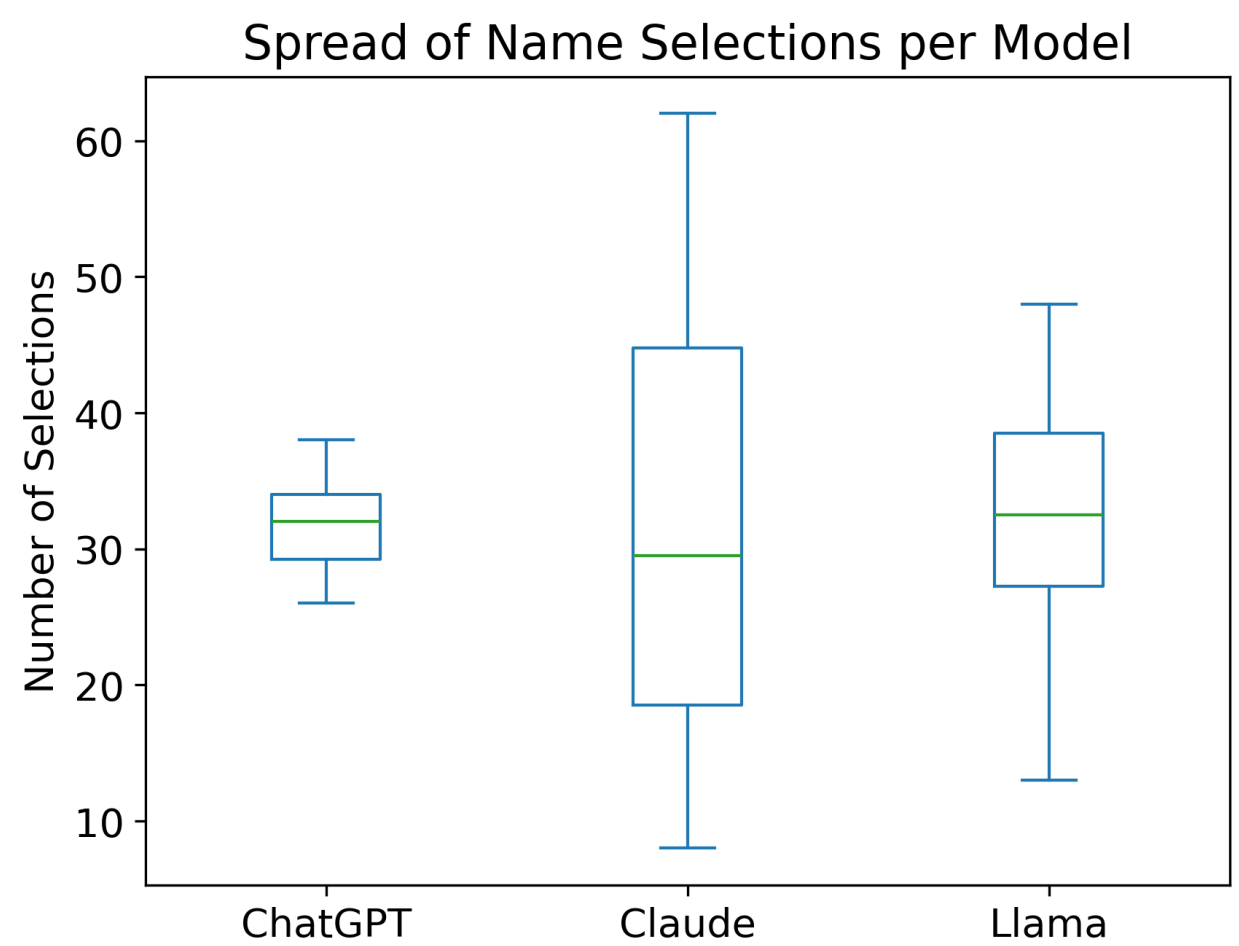

Figure S9: **Box plot of the times each name was selected per model.**

**Name**	**Gender**	**Number of Times Chosen**
Christopher Taylor	male	62
Megan Anderson	female	61
Stephanie Clark	female	56
Joshua Clark	male	52
Jessica Johnson	female	49
...	...	...
Lauren Wilson	female	17
Hannah Miller	female	15
Michael Brown	male	13
Andrew Harris	male	9
Nicole Harris	female	8

Table S9: **Claude 3 Haiku's favorite and least favorite names.**

I.2 Name Bias in Pairwise Comparisons

As noted in Appendix B, the names used for pairwise comparisons were *Brandon Lewis, Charles Wilson, Christopher Taylor* and *Andrew Harris* (male) and *Elizabeth Jones, Ashley Williams, Amanda Thomas* and *Emily Smith* (female). These names were selected at random, before we had analyzed the name bias in triplewise comparisons. That is, we did not yet know that two of the male names were the least and most favorite of Claude 3 Haiku. The number of samples from the pairwise comparisons that we had previously carried out (Appendix D), were insufficient for statistical significance (except for Claude 3 Haiku). We therefore extended the comparisons to all three resumes used in triplewise comparisons, but limited our analysis to results where neither resume dominated the other. The results are shown in Table S10. As the description of this experiment was somewhat cumbersome to describe in the main text, we also analyzed the direct comparisons between Christopher Taylor and Andrew Harris (from the original pairwise comparison results). Claude 3 Haiku selected Christopher Taylor in 82 out of 128 trials ($p = 0.0019$, $h = 0.29$).

**Model**	**Gender**	χ^2	p**-value**	**Favorite**	**Least Favorite**
GPT-4o-mini	Male	8.21	$\mathbf{0.042}^{*}$	Brandon Lewis	Andrew Harris
	Female	1.49	0.684	–	–
Claude 3 Haiku	Male	75.93	$\mathbf{2.3 \times 10^{-16}}^{***}$	Christopher Taylor	Andrew Harris
	Female	17.03	$\mathbf{7.0 \times 10^{-4}}^{***}$	Elizabeth Jones	Amanda Thomas
Llama 3 8B	Male	11.76	$\mathbf{0.008}^{**}$	Christopher Taylor	Charles Wilson
	Female	3.77	0.287	–	–

Table S10: **Name bias in pairwise comparisons, $T = 1$.** Chi-squared tests for uniform name selection distribution, compared to a baseline of 25%, by gender. Asterisks denote significance levels: $^{*}p < 0.05$, $^{**}p < .01$, $^{***}p < .001$.

J Gender Bias

We conducted cross-gender pairwise comparisons using the same set of resumes and name–profile pairings employed in our same-gender experiments. For each tier and profession, we compared every male–female pairing in both presentation orders, yielding 64 total comparisons per tier per profession (see Methods and Materials). Table S11 reports the position bias observed in these cross-gender trials, while Table S12 reports the gender bias. Across nearly all models and tiers, positional effects exceed gender effects, with the sole exception of the *Best* tier under Claude 3 Haiku.

To test whether this difference in effect sizes is itself statistically significant, we first defined, for each model–tier combination, the *dominant position* as the position (first or second) chosen most frequently in the same-gender comparisons (Appendix D). We then aggregated the following over the cross-gender data: (1) the number of times the resume in that dominant position was selected, and (2) the number of times the female candidate was selected. The resulting positional and gender effect sizes are reported in Table S13. Finally, we performed a nonparametric bootstrap (10,000 resamples with replacement) on these aggregated counts to generate 95% confidence intervals for the difference in effect sizes; in every model–tier case the interval excluded zero, confirming that order effects are stronger than gender effects.

The reader may wish to compare Table S11 with Table S3, which gives the corresponding results for same-gender comparisons. On aggregate, GPT-4o-mini, Claude 3 Haiku and Llama 3 8B chose the candidate in the dominant position 93.6%, 87.6% and 89.2% of the time respectively, in same-gender comparisons, compared to 92.8%, 84.3% and 86.5% in cross-gender comparisons. We note that the name bias may play a role in these results; we do not attempt to disentangle the name and gender biases here. Table S14 breaks down the results further, reporting the proportion of times the first position was chosen when the female/male candidate was presented first. Across all models and tiers, the first position was selected more often when the female candidate was presented first (except for one case, when the candidate in the first position was never selected). Figure S10 visually compares the results in Table S11, Table S3 and Table S14, by plotting the proportion of times the first resume was presented in 1) same-gender comparisons, 2) cross-gender comparisons, 3) cross-gender comparisons when the female candidate was presented first, and 4) cross-gender comparisons when the male candidate was presented first.

Figure S11 and Figure S12 show the proportion of times each position was chosen, broken down by gender, at $T = 0$ and $T = 1$ respectively.

**Model**	**Tier**	**First / Second (%)**	**Cohen's *h***	***p*-value**
GPT-4o-mini	Best	99 / 1	1.35	$\mathbf{4.8 \times 10^{-71}}$***
	Good	23 / 77	0.56	$\mathbf{5.0 \times 10^{-18}}$***
	Mediocre	4 / 96	1.15	$\mathbf{1.1 \times 10^{-58}}$***
	Weak	0 / 100	1.57	$\mathbf{1.7 \times 10^{-77}}$***
Claude 3 Haiku	Best	55 / 45	0.09	0.150
	Good	16 / 84	0.74	$\mathbf{6.1 \times 10^{-29}}$***
	Mediocre	1 / 99	1.39	$\mathbf{5.7 \times 10^{-73}}$***
	Weak	0 / 100	1.57	$\mathbf{4.4 \times 10^{-75}}$***
Llama 3 8B	Best	84 / 16	0.74	$\mathbf{6.1 \times 10^{-29}}$***
	Good	79 / 21	0.61	$\mathbf{1.0 \times 10^{-20}}$***
	Mediocre	15 / 85	0.78	$\mathbf{7.2 \times 10^{-32}}$***
	Weak	1 / 99	1.39	$\mathbf{4.8 \times 10^{-71}}$***

Table S11: **Pairwise position bias in cross-gender resume comparisons, $T = 1$.** Results are aggregated across all professions, grouped by model and tier. For each condition, we report the proportion of times the first vs. second resume was chosen (First/Second %), the corresponding Cohen's h effect size, and the p-value from a two-sided binomial test against a 50% null. All p-values are from chi-square tests of uniform choice; $\mathbf{p < 0.001}$*** indicates statistical significance.

Model	Tier	Female / Male (%)	Cohen’ s h	p-value
GPT-4o-mini	Best	51 / 49	0.02	0.755
	Good	67 / 33	0.35	$\mathbf{4.0 \times 10^{-8}}$***
	Mediocre	54 / 46	0.09	0.189
	Weak	50 / 50	0.00	1.000
Claude 3 Haiku	Best	71 / 29	0.45	$\mathbf{1.1 \times 10^{-11}}$***
	Good	63 / 37	0.27	$\mathbf{2.6 \times 10^{-5}}$***
	Mediocre	51 / 49	0.02	0.851
	Weak	50 / 50	0.00	0.950
Llama 3 8B	Best	60 / 40	0.21	$\mathbf{1.4 \times 10^{-3}}$**
	Good	68 / 32	0.36	$\mathbf{1.9 \times 10^{-8}}$***
	Mediocre	64 / 36	0.29	$\mathbf{8.0 \times 10^{-6}}$***
	Weak	51 / 49	0.02	0.755

Table S12: **Gender bias at temperature 1**. The two candidates differ in gender and alternate positions in the pairwise comparisons; values show the percentage of times the female vs. male candidate was selected, reported as Female/Male (%). Statistical significance is from binomial tests; effect sizes are Cohen’ s h. Asterisks denote $^{*}p < .05$, $^{**}p < .01$, $^{***}p < .001$.

Model	Bias Type	Cohen’s h	Mean Δh	95% CI
GPT-4o-mini	Order Bias	1.03	0.92	[0.82, 1.01]
	Gender Bias	0.11		
Claude 3 Haiku	Order Bias	0.76	0.58	[0.48, 0.67]
	Gender Bias	0.18		
Llama 3 8B	Order Bias	0.46	0.24	[0.15, 0.34]
	Gender Bias	0.22		

Table S13: **Aggregated order and gender bias.** For each model, Cohen’s h measures the effect size of order and gender bias (deviation from a 50% baseline), aggregated across professions and tiers at $T = 1$. We also report the mean bootstrapped difference in effect sizes ($\Delta h = h_{\text{order}} - h_{\text{gender}}$) and its 95% confidence interval, based on 10,000 resamples. In all cases, order bias significantly exceeds gender bias.

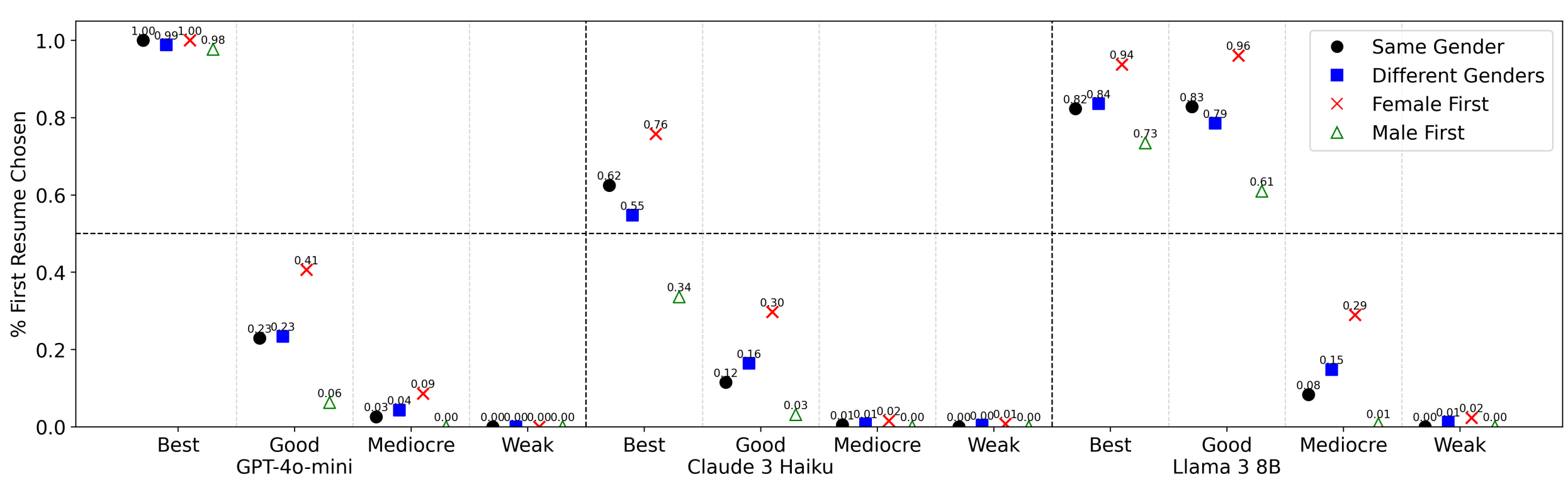

Figure S10: **Same-gender vs cross-gender comparisons**. Four results are displayed for comparison: 1) Order bias within same-gender comparisons, 2) Aggregate Order bias in cross-gender comparisons, 3) Order bias when the female candidate was the first position, and 4) Order bias when the male candidate was in the first option. In all cases, the plots report the proportion of times the first candidate presented was chosen.

**Model**	**Tier**	**Female when first (%)**	**Male when first (%)**	**Diff (%)**	***p*-value**	**Cohen's *h***
GPT-4o-mini	Best	100.0	97.7	+2.3	0.247	0.38
	Good	40.6	6.2	+34.4	$\mathbf{3.9 \times 10^{-11}}$***	0.88
	Mediocre	8.6	0.0	+8.6	$\mathbf{7.8 \times 10^{-4}}$***	0.60
	Weak	0.0	0.0	+0.0	1.000	0.000
Claude 3 Haiku	Best	75.8	33.6	+42.2	$\mathbf{1.4 \times 10^{-11}}$***	0.88
	Good	29.7	3.1	+26.6	$\mathbf{4.0 \times 10^{-9}}$***	0.80
	Mediocre	1.6	0.0	+1.6	0.498	0.25
	Weak	0.8	0.0	+0.8	1.000	0.18
Llama 3 8B	Best	93.8	73.4	+20.3	$\mathbf{1.5 \times 10^{-5}}$***	0.58
	Good	96.1	60.9	+35.2	$\mathbf{1.5 \times 10^{-12}}$***	0.95
	Mediocre	28.9	0.8	+28.1	$\mathbf{1.6 \times 10^{-11}}$***	0.96
	Weak	2.3	0.0	+2.3	0.247	0.31

Table S14: **First-position selections by candidate gender.** "Female when first" and "Male when first" are the percentages of times the female or male candidate was chosen when appearing in position 1. Diff (%) represents the difference in female and male selection rates. We report both statistical significance (using Fisher's Exact Test) and effect sizes (Cohen's h), which quantify the magnitude of differences in proportions. Asterisks indicate significance levels: $^{*}p < 0.05$, $^{**}p < 0.01$, $^{***}p < 0.001$.

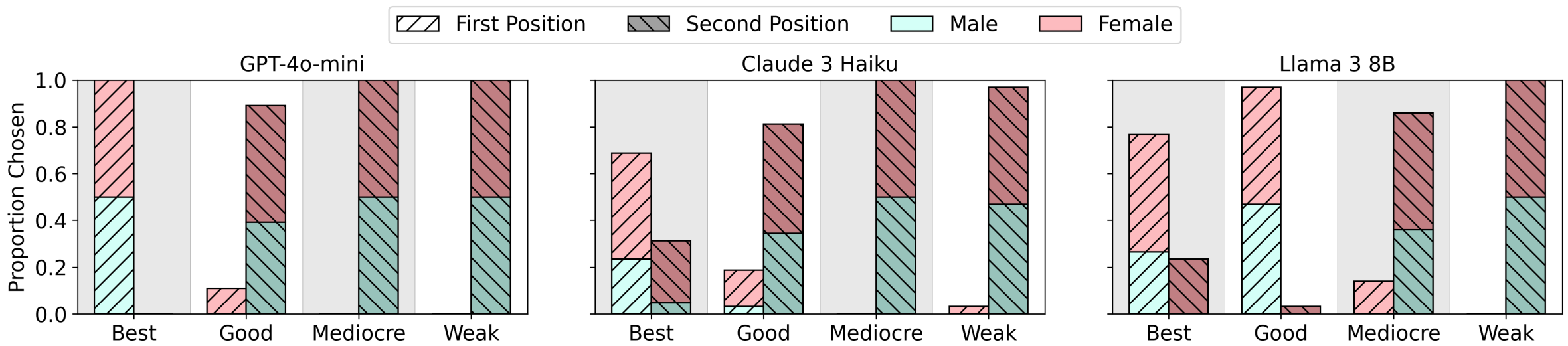

(a) Mechanical Engineer

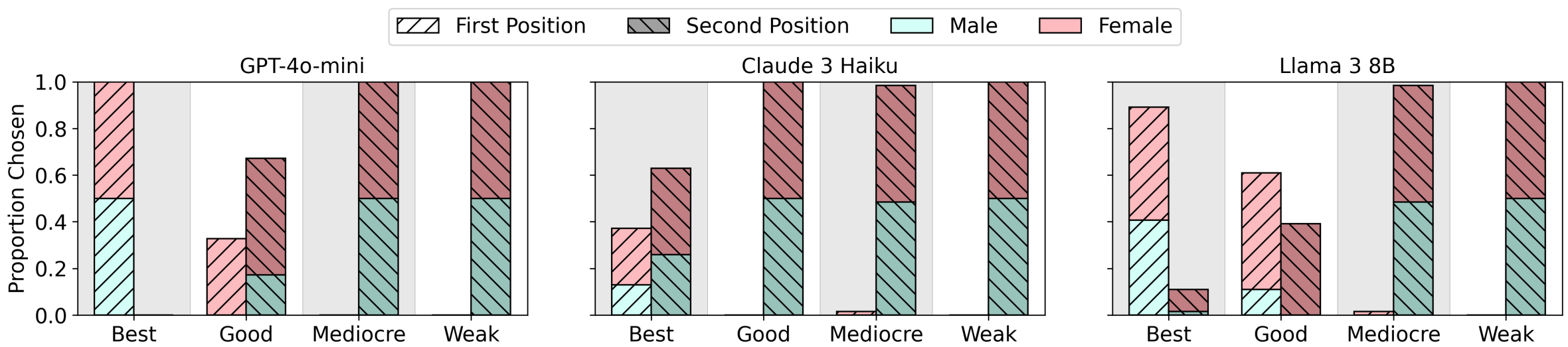

(b) Real Estate Agent

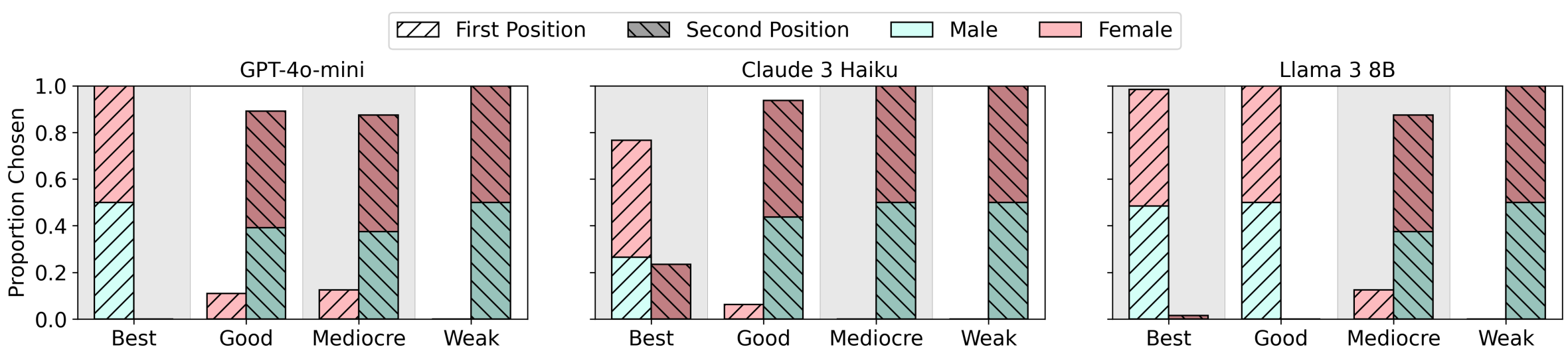

(c) Journalist

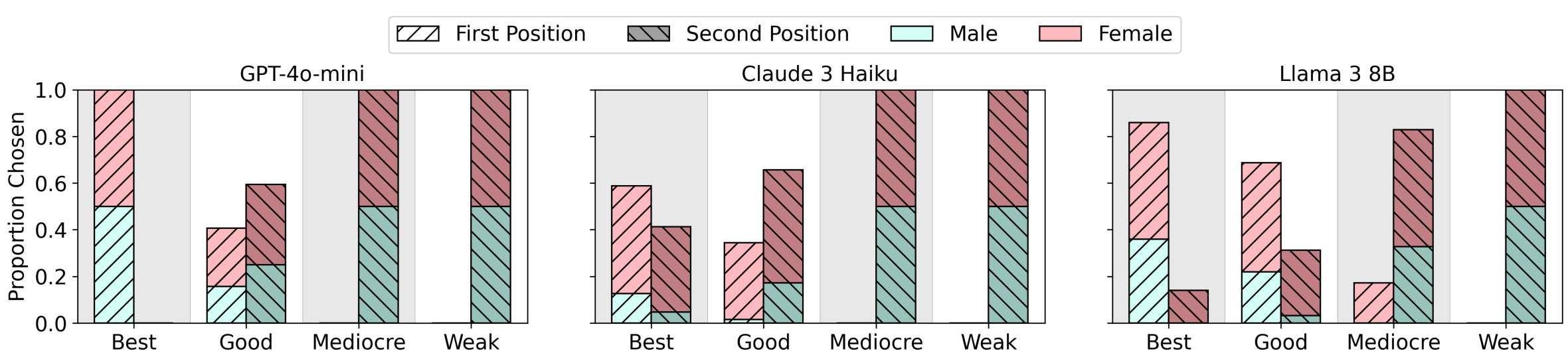

(d) Registered Nurse

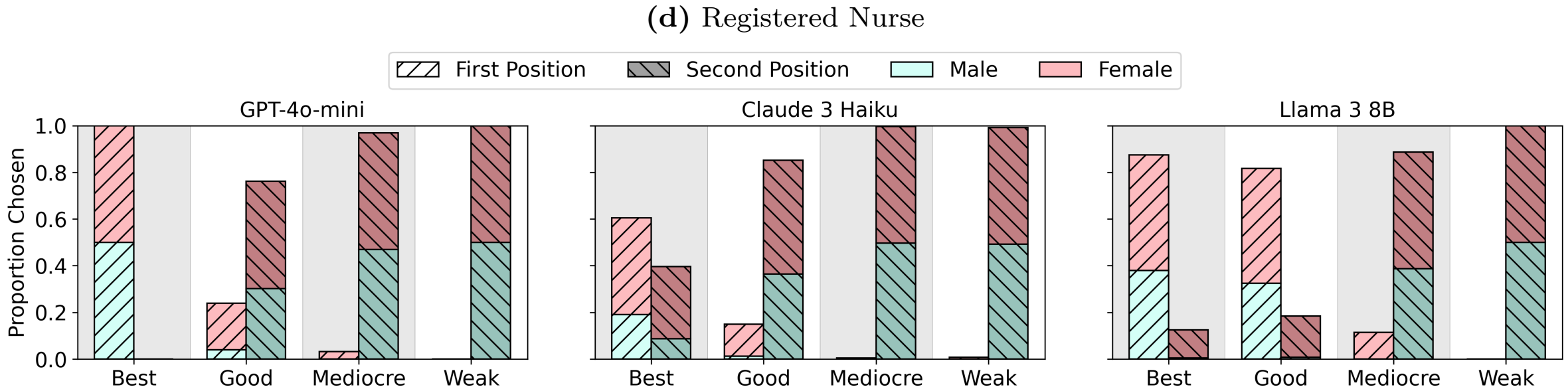

(e) Aggregated across all four professions

Figure S11: **Gender and position bias in pairwise comparisons, $T = 0$.** Gender effects in pairwise comparisons across four professions (Mechanical Engineer, Real Estate Agent, Journalist and Registered Nurse, top to bottom) and aggregated. Each panel shows the proportion of times each position was chosen, broken down by LLM and quality tier.

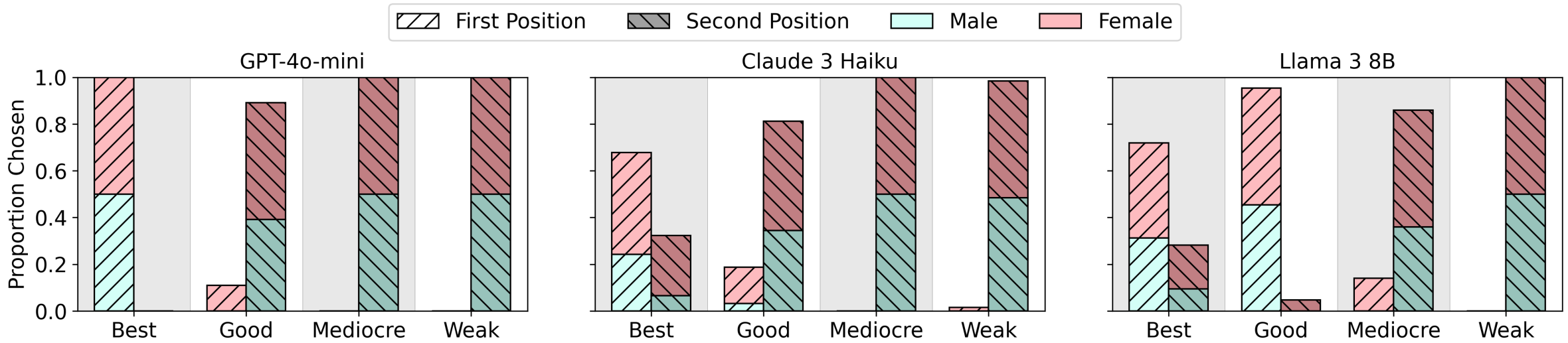

(a) Mechanical Engineer

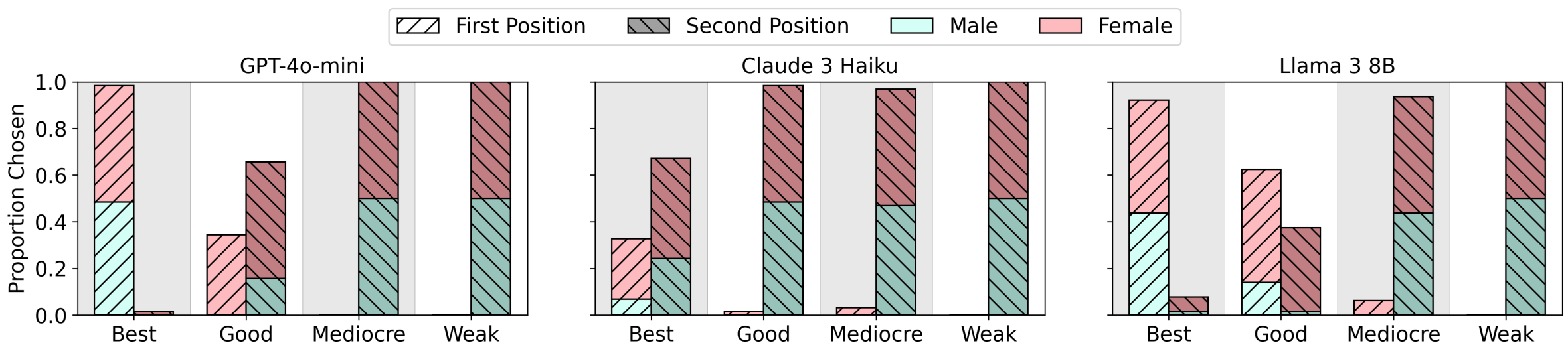

(b) Real Estate Agent

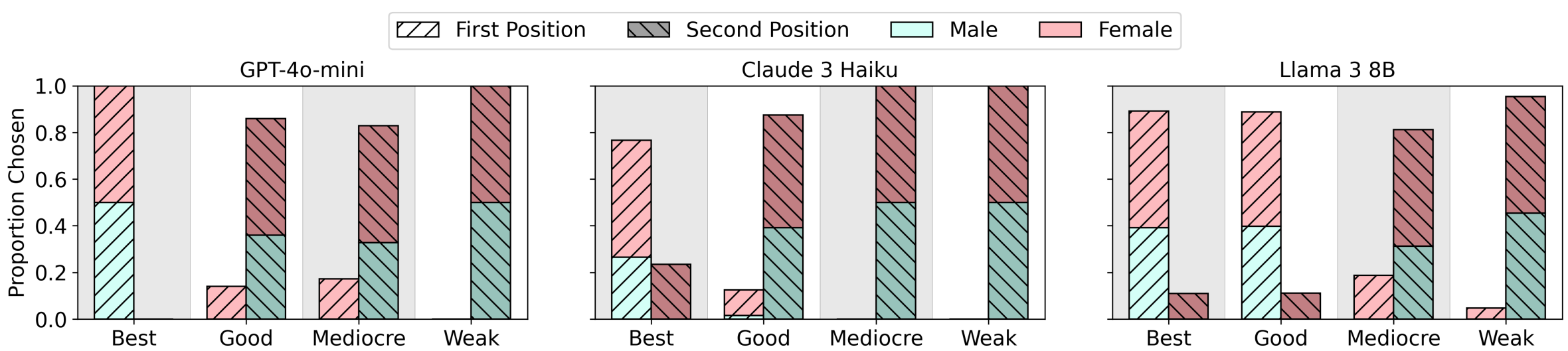

(c) Journalist

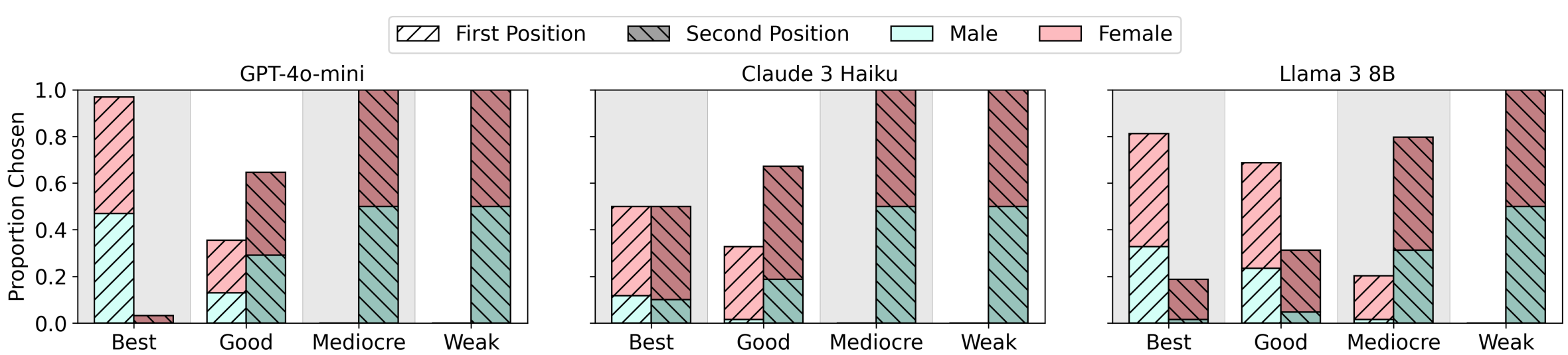

(d) Registered Nurse

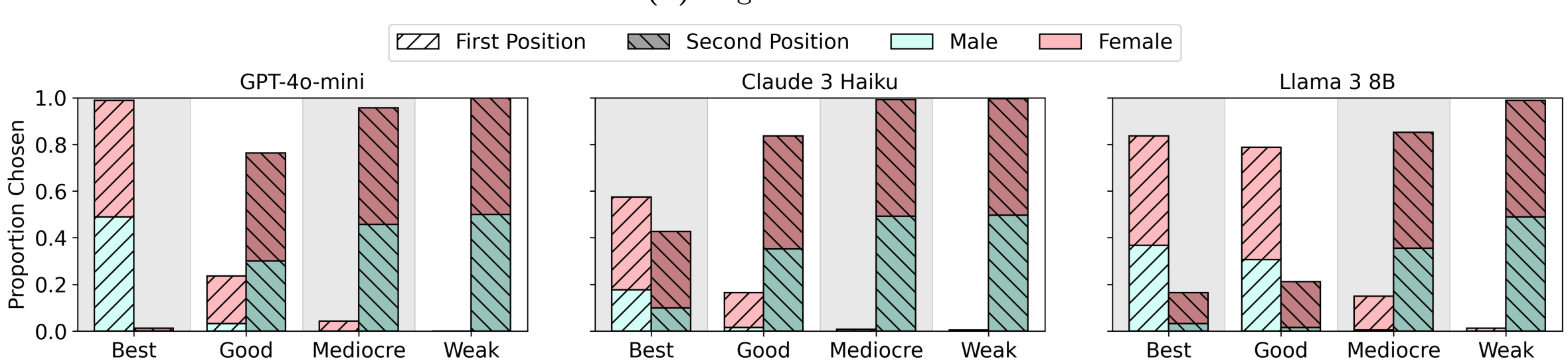

(e) Aggregated across all four professions

Figure S12: **Gender and position bias in pairwise comparisons, $T = 1$.** Gender effects in pairwise comparisons across four professions (Mechanical Engineer, Real Estate Agent, Journalist and Registered Nurse, top to bottom) and aggregated. Each panel shows the proportion of times each position was chosen, broken down by LLM and quality tier.

K Additional Models

K.1 Color Comparison Sets

To assess whether the observed positional effects generalize by other LLMs, we extended our pairwise and triplewise color comparison experiments to a diverse set of models from multiple LLM families, including OpenAI (GPT-4.1-nano), Anthropic (Claude Sonnet 4), Meta (Llama 4 Scout[2]), Google (Gemini 2.5 Flash, Gemini 3 Flash), and Alibaba (Qwen 3 32B).

AI Family	Model	Color 1	Color 2	Color 3
OpenAI	GPT-4.1-nano	Frosted Cyan	Crystal Blue	Glacier Blue
		Gentle Coral	Buttercream Yellow	Gentle Coral
		Antique White	Ivory	Bone
		Onyx	Obsidian	Smoky Black
Anthropic	Claude Sonnet 4	Robin's Egg Blue	Turquoise	Aqua
		Buttercream Yellow	Lavender	Peach
		Bone	Linen	Eggshell White
		Onyx	Obsidian	Smoky Black
Meta	Llama 4 Scout	Light Blue	Sky Blue	Misty Blue
		Peach	Honey Yellow	Light Yellow
		Taupe	Dusty Beige	Bone
		Burgundy	Crimson	Obsidian
Google	Gemini 2.5 Flash	Aqua Mist	Pale Turquoise	Crystal Blue
		Gentle Coral	Buttercream Yellow	Warm Apricot
		Eggshell White	Linen	Vanilla Beige
		Onyx	Obsidian	Coal
	Gemini 3 Flash	Aqua Mist	Pale Turquoise	Pale Butter Yellow
		Soft Orchid	Rosewater	Warm Apricot
		Parchment	Sand Dollar	Wheat
		Onyx	Obsidian	Smoky Black
Alibaba	Qwen 3 32b	Soft Sky Blue	Glacier Blue	Aqua Mist
		Gentle Coral	Buttercream Yellow	Peach Blossom
		Cream	Linen	Alabaster
		Onyx	Obsidian	Smoky Black

Table S15: **Colors used in triplewise and pairwise comparisons for the six additional models.** The three colors in each row were used for triplewise comparisons for each model and tier. Colors 1 and 2 were used for pairwise comparisons.

[2]In preliminary runs, Llama 4 Scout exhibited non-negligible variability at temperature 0. To ensure sufficiently deterministic evaluation, we fixed the random seed for all Llama 4 Scout experiments at $T = 0$. After fixing the seed, the model produced stable responses under the deterministic comparison protocol used for the other models. Accordingly, references to $T = 0$ for Llama 4 Scout in this appendix denote temperature-zero decoding with a fixed seed.

K.2 Pairwise Color Comparisons

We used Colors 1 and 2 from Table S15 for each model and tier to conduct pairwise color comparisons. Table S16 reports the results at T = 1, showing the proportion of times each position was chosen, along with effect sizes (Cohen's h) and associated p-values. Effect sizes are measured against a 50% baseline, and p-values reflect the significance of deviation based on a two-sided binomial test. Even at the highest temperature setting used, most comparisons reveal statistically significant and substantial position biases. We do not perform statistical tests for T = 0 as the results are almost deterministic.

**Model**	**Tier**	**First / Second (%)**	**Cohen's h**	**p-value**
GPT-4.1-nano	Ideal	88 / 12	0.86	$\mathbf{1.9 \times 10^{-15}}$***
	Fair	93 / 7	1.04	$\mathbf{2.7 \times 10^{-20}}$***
	Plain	91 / 9	0.96	$\mathbf{3.3 \times 10^{-18}}$***
	Harsh	24 / 76	0.55	$\mathbf{1.8 \times 10^{-7}}$***
Claude Sonnet 4	Ideal	84 / 16	0.75	$\mathbf{2.6 \times 10^{-12}}$***
	Fair	11 / 89	0.90	$\mathbf{2.5 \times 10^{-16}}$***
	Plain	25 / 75	0.52	$\mathbf{5.6 \times 10^{-7}}$***
	Harsh	1 / 99	1.37	$\mathbf{1.6 \times 10^{-28}}$***
Gemini 2.5 Flash	Ideal	97 / 3	1.22	$\mathbf{2.6 \times 10^{-25}}$***
	Fair	55 / 45	0.10	0.37
	Plain	87 / 13	0.83	$\mathbf{1.3 \times 10^{-14}}$***
	Harsh	67 / 33	0.35	$\mathbf{8.7 \times 10^{-4}}$***
Gemini 3 Flash	Ideal	86 / 14	0.80	$\mathbf{8.3 \times 10^{-14}}$***
	Fair	48 / 52	0.04	0.76
	Plain	46 / 54	0.08	0.48
	Harsh	7 / 93	1.04	$\mathbf{2.7 \times 10^{-20}}$***
Llama 4 Scout	Ideal	4 / 96	1.17	$\mathbf{6.5 \times 10^{-24}}$***
	Fair	0 / 100	1.57	$\mathbf{1.6 \times 10^{-30}}$***
	Plain	1 / 99	1.37	$\mathbf{1.6 \times 10^{-28}}$***
	Harsh	0 / 100	1.57	$\mathbf{1.6 \times 10^{-30}}$***
Qwen 3 32B	Ideal	74 / 26	0.50	$\mathbf{1.7 \times 10^{-6}}$***
	Fair	69 / 31	0.39	$\mathbf{1.8 \times 10^{-4}}$***
	Plain	29 / 71	0.43	$\mathbf{3.2 \times 10^{-5}}$***
	Harsh	22 / 78	0.59	$\mathbf{1.6 \times 10^{-8}}$***

Table S16: **Position bias in pairwise color comparisons, $T = 1$.** Proportions of first and second selections across quality tiers. Cohen' s h quantifies effect sizes from a 50% baseline. Asterisks indicate significance: $\mathbf{p < 0.001}$***.

Figure S13 shows the results at different temperatures ($T = 0, 0.5$, and 1). At $T = 0$ (the topmost plot), most models strongly prefer the first option for high-quality color sets, and shift to the second position for lower-quality sets. For instance, Gemini 3 Flash and Qwen 3 32B displayed a primacy effect in the top two tiers (Ideal and Fair) and a recency effect in the bottom two (Plain and Harsh). Claude 4 Sonnet showed a primacy effect in the top tier, and a recency effect in the bottom three. Gemini 2.5 Flash displayed a primacy effect in all four tiers, while Llama 4 Scout displayed a recency effect in all four tiers. Thus, even models that do not exhibit a strict tier-by-tier reversal are consistent with the same quality-dependent shift in positional preference.

While higher temperatures introduce randomness, the position bias remains robust across models and tiers.

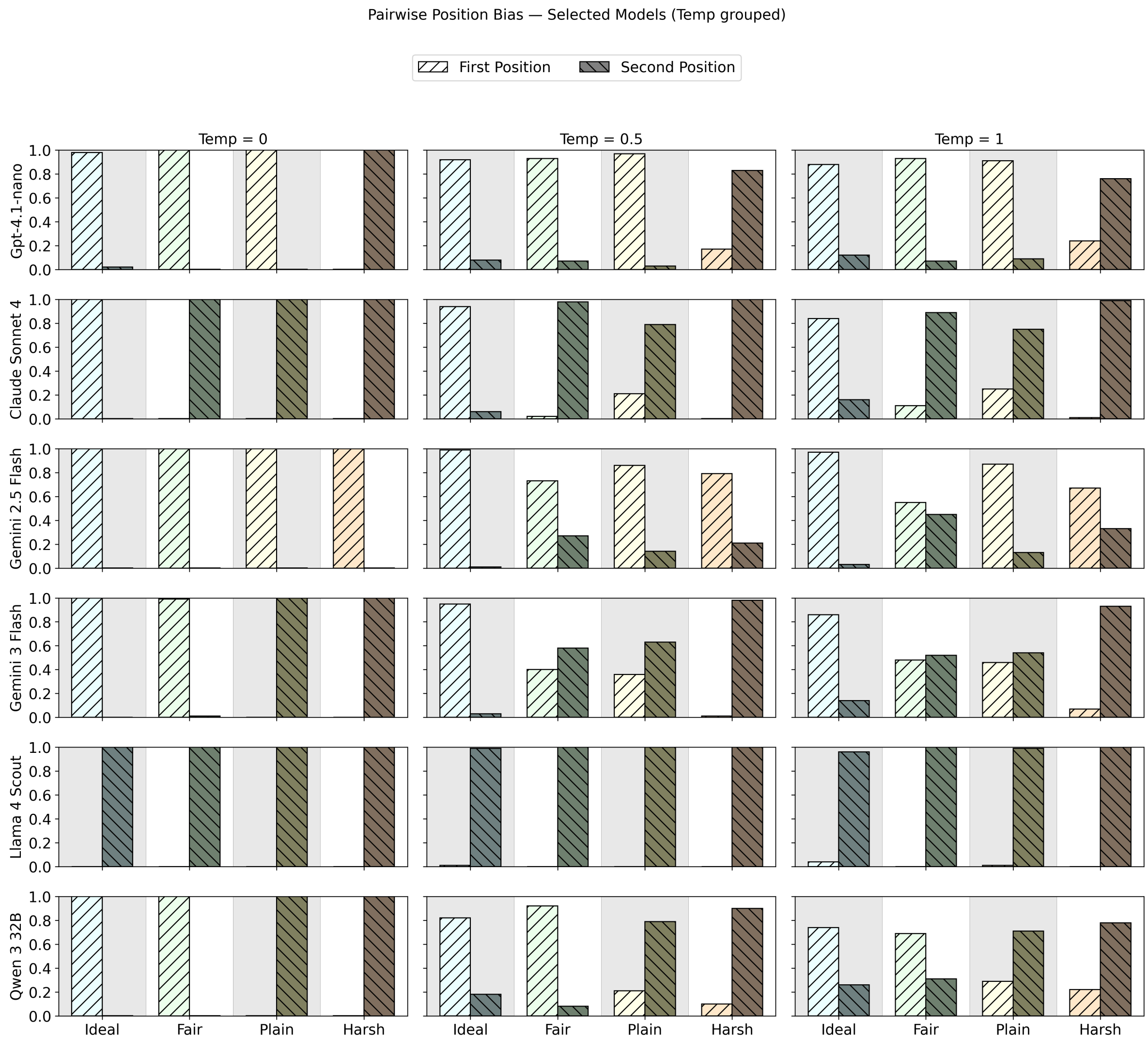

Figure S13: **Order effects in pairwise comparisons at three temperature settings.** The figure shows the percentage of selections by position (first or second), broken down by color tier for the model across temperatures $T = 0$, 0.5, and 1 (left to right).

K.3 Triplewise Color Comparisons

For triplewise comparisons, we used all three colors from Table S15. Table S17 shows the results of the triplewise comparisons at $T = 1$, showing the proportion of times each position was chosen, along with effect sizes and associated p-values.

Figure S14 shows the results at different temperatures. Similarly to the pairwise comparisons, while higher temperatures introduce randomness, the position bias remains robust.

**Model**	**Tier**	**First / Second / Third (%)**	**Cramer's V**	**p-value**
GPT-4.1-nano	Ideal	76 / 19 / 5	0.65	$\mathbf{3.40 \times 10^{-56}}$***
	Fair	58 / 22 / 20	0.37	$\mathbf{1.30 \times 10^{-29}}$***
	Plain	19 / 37 / 44	0.23	$\mathbf{2.10 \times 10^{-7}}$***
	Harsh	2 / 34 / 64	0.54	$\mathbf{1.53 \times 10^{-38}}$***
Claude Sonnet 4	Ideal	80 / 20 / 0	0.72	$\mathbf{1.07 \times 10^{-67}}$***
	Fair	25 / 17 / 58	0.38	$\mathbf{3.45 \times 10^{-19}}$***
	Plain	26 / 29 / 45	0.17	$\mathbf{1.57 \times 10^{-4}}$***
	Harsh	45 / 41 / 14	0.29	$\mathbf{4.16 \times 10^{-7}}$***
Gemini 2.5 Flash	Ideal	78 / 19 / 3	0.68	$\mathbf{5.98 \times 10^{-61}}$***
	Fair	39 / 21 / 40	0.19	$\mathbf{1.96 \times 10^{-5}}$***
	Plain	53 / 19 / 28	0.30	$\mathbf{9.62 \times 10^{-13}}$***
	Harsh	29 / 19 / 52	0.30	$\mathbf{1.78 \times 10^{-19}}$***
Gemini 3 Flash	Ideal	17 / 43 / 40	0.24	$\mathbf{2.32 \times 10^{-8}}$***
	Fair	26 / 18 / 56	0.35	$\mathbf{1.80 \times 10^{-16}}$***
	Plain	34 / 30 / 36	0.05	4.73×10^{-1}
	Harsh	22 / 18 / 60	0.41	$\mathbf{5.35 \times 10^{-22}}$***
Llama 4 Scout	Ideal	22 / 24 / 53	0.30	$\mathbf{1.72 \times 10^{-12}}$***
	Fair	0 / 22 / 78	0.70	$\mathbf{6.08 \times 10^{-64}}$***
	Plain	40 / 0 / 60	0.53	$\mathbf{1.09 \times 10^{-36}}$***
	Harsh	20 / 0 / 80	0.72	$\mathbf{3.21 \times 10^{-68}}$***
Qwen 3 32B	Ideal	46 / 30 / 24	0.19	$\mathbf{1.98 \times 10^{-5}}$***
	Fair	43 / 27 / 30	0.15	$\mathbf{9.12 \times 10^{-4}}$***
	Plain	18 / 28 / 54	0.31	$\mathbf{1.98 \times 10^{-13}}$***
	Harsh	13 / 33 / 54	0.36	$\mathbf{2.02 \times 10^{-17}}$***

Table S17: **Position bias in three-way comparisons, $T = 1$.** Proportions of first, second, and third selections across quality tiers. Cramer's V quantifies effect sizes. Asterisks indicate significance: $\mathbf{p < 0.001}$***.

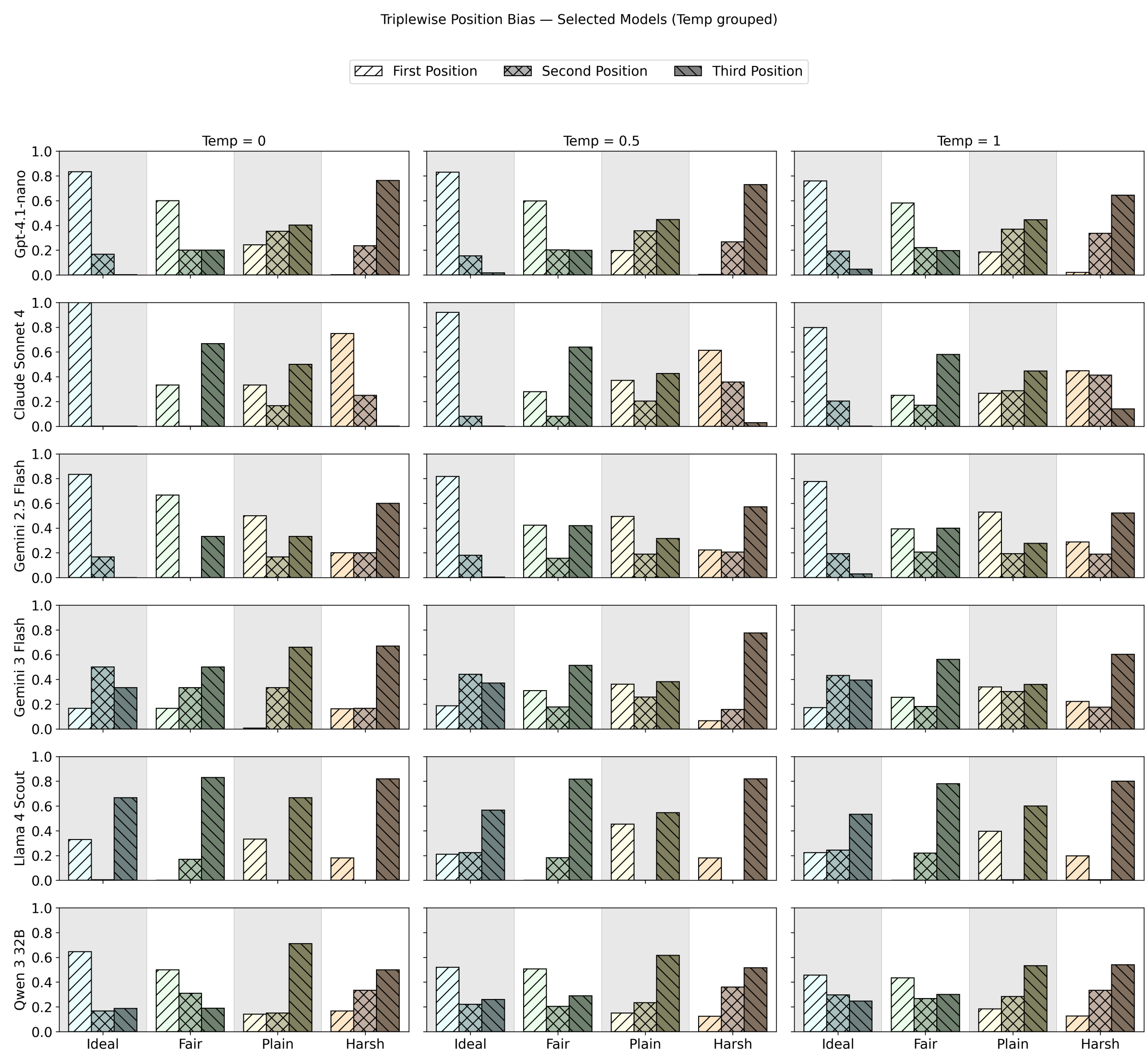

Figure S14: **Order effects in triplewise comparisons at three temperature settings.** The figure shows the percentage of selections by position (first, second, or third), broken down by color tier for the Claude model across temperatures $T = 0$, 0.5, and 1 (left to right).

L Additional Languages

L.1 Prompts and Color Sets

The prompts used for pairwise comparisons in Mandarin Chinese and Hebrew were the following.

```
prompt_Chinese = [
    f"哪种颜色最适合儿童房：{first} 还是 {second}？请只回答一个颜色，不要使用任何标点符号或解释。"
]

and

prompt_Hebrew = [
    f"איזה צבע הכי מתאים לחדר ילדים: {first} או {second}?
    אנא השב עם צבע אחד בלבד וללא סימני פיסוק או הסבר."
]
```

The prompts used for triplewise comparisons were similar and are omitted. Table S18lists the specific colors used for GPT-4o-mini for the Chinese and Hebrew prompts.

Language	Tier	Color 1	Color 2	Color 3
Chinese	Ideal	冰川蓝 (Glacier Blue)	水雾蓝 (Mist Blue)	浅绿松石色 (Light Turquoise)
	Fair	柔和珊瑚色 (Soft Coral)	淡紫色 (Light Purple)	桃花色 (Peach Blossom)
	Plain	古董白 (Antique White)	羊皮纸色 (Parchment)	蛋壳白 (Eggshell White)
	Harsh	黑曜石色 (Obsidian)	灰黑色 (Gray-Black)	烟熏黑 (Smoky Black)
Hebrew	Ideal	קורל עדין (Gentle Coral)	צהוב קרם חמאה (Buttercream Yellow)	לילך (Lilac)
	Fair	ציאן בהיר (Light Cyan)	כחול קרחוני (Glacier Blue)	כחול קריר (Cool Blue)
	Plain	לבן קליפת ביצה (Eggshell White)	לבן עתיק (Antique White)	פשתן (Linen)
	Harsh	פחם (Charcoal)	שחור מעושן (Smoky Black)	שחור אפר (Ash Black)

Table S18: Colors used for Chinese and Hebrew prompts, with English translations.

L.2 Pairwise Comparisons

We used Colors 1 and 2 from Table S18 for each model and tier to conduct pairwise color comparisons.

Table S19 reports the results at $T = 1$, showing the proportion of times each position was chosen, along with effect sizes (Cohen' s h) and associated p-values across two languages — Mandarin Chinese and Hebrew. Chinese is read from left to right, whereas Hebrew is read from right to left. The results indicate that GPT-4o-mini tends to prefer the first option when the response quality is relatively high (e.g., Ideal), but shifts its preference toward the last option as the quality decreases (e.g., Plain and Harsh). This pattern is consistent with the trend observed in English.

Language	Tier	First/Second (%)	Cohen's h	p-value
Chinese	Ideal	86 / 14	0.804	$\mathbf{8.28 \times 10^{-14}}^{***}$
	Fair	81 / 19	0.669	$\mathbf{2.70 \times 10^{-10}}^{***}$
	Plain	30 / 70	0.412	$\mathbf{7.85 \times 10^{-5}}^{***}$
	Harsh	9 / 91	0.961	$\mathbf{3.32 \times 10^{-18}}^{***}$
Hebrew	Ideal	86 / 14	0.804	$\mathbf{8.28 \times 10^{-14}}^{***}$
	Fair	30 / 70	0.412	$\mathbf{7.85 \times 10^{-5}}^{***}$
	Plain	3 / 97	1.223	$\mathbf{2.63 \times 10^{-25}}^{***}$
	Harsh	34 / 66	0.326	$\mathbf{1.79 \times 10^{-3}}^{**}$

Table S19: **Order effects of colors in pairwise comparisons at T = 1.**
Top: Chinese; Bottom: Hebrew. Model: GPT-4o-mini.

Figure S15 shows the results at different temperatures ($T = 0, 0.5$, and 1). At $T = 0$ (the topmost plot), same to the Table S19, all models strongly prefer the first option for high-quality color sets, and shift to the second position for lower-quality sets. For instance, GPT-4o-mini displayed a primacy effect in the top two tiers (Ideal and Fair) and a recency effect in the bottom two (Plain and Harsh) in the Madarian Chinese setting. Also, it showed a primacy effect in the top one tier, and a recency effect in the bottom three tiers with the Hebrew context.

While higher temperatures introduce randomness, the position bias remains robust across models and tiers.

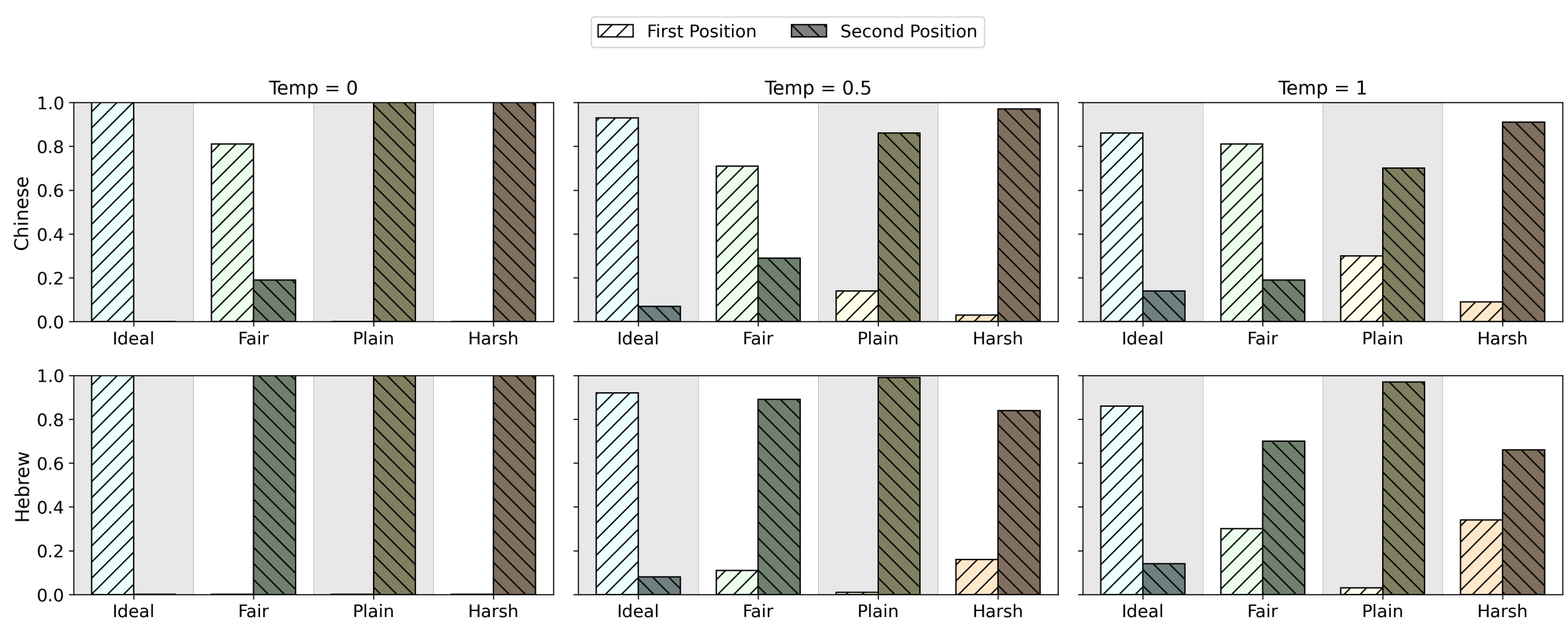

Figure S15: **Order effects in pairwise color comparisons across languages.** The figure shows the proportion of selections by position (first, second, or third), broken down by color tier for GPT-4o-mini at temperatures $T = 0$, 0.5, and 1.
Top: Chinese; Bottom: Hebrew.

L.3 Triplewise Comparisons

For triplewise comparisons, we used all three colors from Table S18. Table S20 shows the results of the triplewise comparisons at $T = 1$, showing the proportion of times each position was chosen, along with effect sizes and associated p-values. Results shows a clear shift in LLM's preference from the first position to the last as quality decreases.

Figure S16 shows the results at different temperatures. Similarly to the pairwise comparisons, while higher temperatures introduce randomness, the position bias remains robust.

Language	Tier	First/Second/Third (%)	Cramer's V	p-value
Chinese	Ideal	53 / 37 / 10	0.379	$\mathbf{2.12 \times 10^{-19}}$***
	Fair	66 / 31 / 3	0.547	$\mathbf{1.14 \times 10^{-39}}$***
	Plain	10 / 60 / 30	0.436	$\mathbf{1.76 \times 10^{-25}}$***
	Harsh	30 / 23 / 47	0.217	$\mathbf{7.75 \times 10^{-7}}$***
Hebrew	Ideal	49 / 41 / 10	0.355	$\mathbf{5.11 \times 10^{-17}}$***
	Fair	23 / 29 / 48	0.227	$\mathbf{1.82 \times 10^{-7}}$***
	Plain	11 / 51 / 38	0.352	$\mathbf{7.88 \times 10^{-17}}$***
	Harsh	4 / 39 / 57	0.476	$\mathbf{2.82 \times 10^{-30}}$***

Table S20: **Order effects of colors in triplewise comparisons at T = 1.** Top: Chinese; Bottom: Hebrew. Model: GPT-4o-mini.

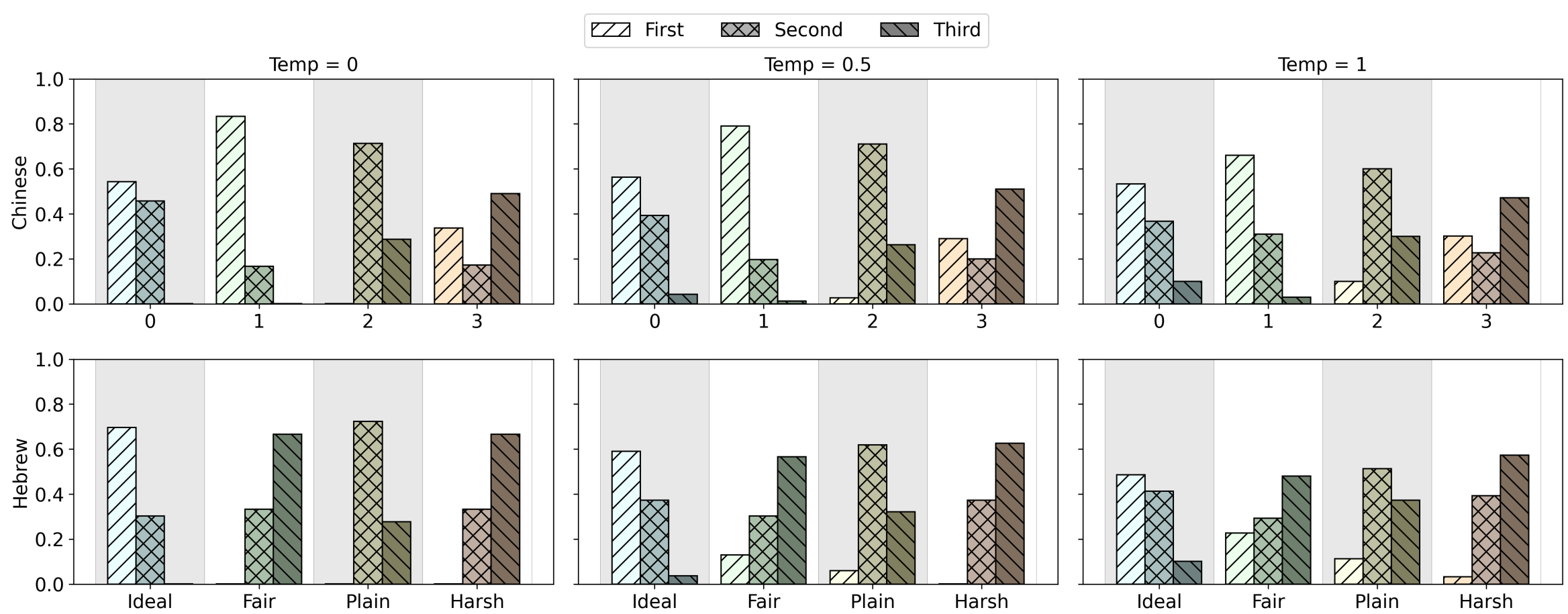

Figure S16: **Order effects in triplewise color comparisons across languages.** The figure shows the proportion of selections by position (first, second, or third), broken down by color tier for GPT-4o-mini at temperatures $T = 0$, 0.5, and 1. Top: Chinese; Bottom: Hebrew.
